# The acute effect of sprint interval training on the immune system: a systematic review and meta-regression analysis

**DOI:** 10.3389/fmed.2025.1703829

**Published:** 2026-02-02

**Authors:** Weibao Liang, Yiqiang Wang, Tianyuan Yu, Yu Hou, Shuting Xu, Zikun Lyu, Yasong Zhang, Wenbai Huang

**Affiliations:** 1 Guangdong Provincial Key Laboratory of Speed Capability Research, Su Bingtian Center for Speed Research and Training, School of Physical Education, Jinan University, Guangzhou, China; 2 School of Sports Training, Xi’an Physical Education University, Xi’an, China; 3 School of Information Technology, Vitebsk State University named after P. M. Masherov, Vitebsk, Belarus; 4 Department of Physical Education, Kunsan National University, Gunsan, Republic of Korea; 5 The First Affiliated Hospital of Jinan University, Guangzhou, China; 6 Department of Basic Medicine, Langfang Health Vocational College, Langfang, China

**Keywords:** acute, cytokines, effect, exercise, immunity, immunology, interval, leukocytes

## Abstract

**Background:**

Sprint interval training (SIT) is an increasingly popular time-efficient training paradigm; however, its acute impact on the immune system remains ambiguous due to inconsistent findings across studies. This systematic review and meta-analysis aimed to quantitatively evaluate the acute effects of a single SIT session on key immunological markers in healthy individuals.

**Methods:**

We systematically searched PubMed, Web of Science, SPORTDiscus, the Cochrane Library, Embase, and Scopus for experimental studies assessing acute immunological changes following a single bout of SIT in healthy participants. Pooled standardized mean differences (SMDs) and 95% confidence intervals (CIs) were calculated using a random-effects model. Non-linear meta-regression and subgroup analyses were performed to investigate sources of heterogeneity, and the certainty of evidence was evaluated using the Grading of Recommendations Assessment, Development and Evaluation (GRADE) methodology.

**Results:**

A total of 21 studies, comprising 359 participants, were included in the analysis. The meta-analysis analysis revealed that a single session of SIT induced a robust and significant increase in total leukocyte count [SMD = 2.68, 95% CI (1.79, 3.57)] and neutrophil count [SMD = 1.04, 95% CI (0.75, 1.34)], with the latter exhibiting zero heterogeneity (*I*^2^ = 0%). Lymphocyte count [SMD = 3.83, 95% CI (1.07, 6.59)] also increased significantly, showing a U-shaped dose–response relationship with repetition duration. Interleukin-6 (IL-6) increased significantly [SMD = 1.10, 95% CI (0.49, 1.71)], with subgroup analysis revealing a twofold greater response in trained athletes compared to untrained individuals. In contrast, salivary IgA (sIgA) remained stable in trained athletes [SMD = 0.07, 95% CI (−0.31, 0.45), *I*^2^ = 0%] but showed high variability in untrained groups. Plasma interleukin-10 (IL-10) concentration exhibited a small, non-significant increasing trend [SMD = 0.18, 95% CI (−0.06, 0.43)].

**Conclusion:**

A single session of SIT elicits a potent acute systemic immune response, characterized by a highly conserved mobilization of circulating leukocytes, particularly neutrophils. This response is significantly modulated by individual training status: athletes exhibit a distinct immunometabolic profile characterized by a robust IL-6 release and stable mucosal immunity (sIgA), whereas untrained individuals show more variable responses. These findings provide critical evidence for understanding the physiological stress of SIT and can inform training and recovery practices to safeguard immune health.

**Systematic review registration:**

https://www.crd.york.ac.uk/PROSPERO/view/CRD420251140621.

## Graphical abstract

**Figure:**
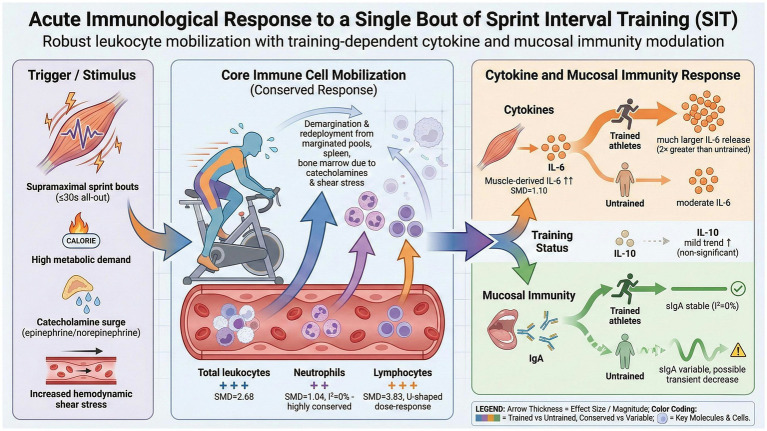
This graphical abstract summarizes the main findings regarding the acute effects of a single session of sprint interval training (SIT) on immune cells, cytokines, and mucosal immunity. Created in BioRender. Liang, W. (2026) https://BioRender.com/9ruwz3p.

## 1 Introduction

Exercise immunology is a foundational discipline for understanding the intricate relationship between physical activity and the immune system. It is well established that regular, moderate-intensity physical exercise confers long-term benefits to immune health, the hallmarks of which include reduced chronic inflammation, enhanced immunosurveillance, and a lower risk of certain infectious diseases (1, 2). In contrast, a single bout of prolonged, high-intensity, or exhaustive exercise can lead to a transient dysregulation of immune function (1, 3, 4). This phenomenon, originally conceptualized as the “open-window” theory, posits that for several hours following strenuous exertion, the body’s immunological defenses—particularly at mucosal surfaces and within the cellular compartment—are temporarily compromised, potentially increasing susceptibility to pathogen invasion and infection (2).

Recently, sprint interval training (SIT) has garnered considerable attention within both competitive sports and public health domains as a highly time-efficient training paradigm. SIT is characterized by brief (typically ≤30 s), repeated bouts of “all-out” or supramaximal intensity exercise, interspersed with relatively long recovery periods (5). A substantial body of evidence has demonstrated that, compared to traditional moderate-intensity continuous training, SIT can elicit similar, if not superior, physiological adaptations—such as improvements in maximal oxygen uptake, anaerobic capacity, blood pressure, and insulin sensitivity—in a fraction of the total training time (6, 7).

Despite its evident benefits, the “all-out” nature inherent to SIT imposes a significant homeostatic challenge, rendering its acute impact on the immune system a critical scientific question. A heterogeneous body of evidence has emerged from individual studies exploring this topic from various perspectives. For instance, some investigations have focused on the dynamic changes in circulating leukocytes and their subsets following SIT (8), while others have examined the response of key plasma cytokines such as IL-6 and IL-10 (9) or assessed alterations in mucosal immunity (10). Although systematic reviews (11) have addressed the broader category of interval training, a quantitative synthesis (i.e., meta-analysis) specifically targeting SIT and its comprehensive effects across various immunological domains is currently lacking, making a clear and holistic understanding of its acute immunological stress elusive.

Therefore, the primary objective of this study was to conduct the first systematic review and meta-analysis to quantitatively synthesize the existing literature on the acute effects of a single SIT session on key immunological markers in healthy individuals. Specifically, our aims were two-fold: (1) to determine the overall effect size of SIT on circulating immune cell counts (total leukocytes, neutrophils, and lymphocytes), plasma cytokine concentrations (IL-6 and IL-10), and mucosal immunity markers (salivary IgA) and (2) to explore, via subgroup analyses and meta-regression analyses, whether participant characteristics (specifically training status) and SIT protocol design (e.g., repetition duration) are key moderators responsible for the heterogeneity across studies. The findings of this investigation will provide crucial evidence for understanding the physiological underpinnings of SIT and will help to optimize training and recovery strategies to safeguard the immune health of the exercising population.

## 2 Methods

### 2.1 Protocol and registration

This systematic review and meta-analysis was designed and reported in accordance with the Preferred Reporting Items for Systematic Reviews and Meta-Analyses (PRISMA) 2020 statement (12). The study protocol was prospectively registered with the International Prospective Register of Systematic Reviews (PROSPERO), registration number CRD420251140621.

### 2.2 Eligibility criteria

Studies were included in this meta-analysis if they met the following pre-specified Population, Intervention, Comparison, Outcomes, and Study Design (PICOS) criteria:

- Population (P): Healthy individuals, without restrictions on age, sex, or training status (including athletes, recreationally active, and sedentary individuals).
- Intervention (I): A single, acute session of sprint interval training (SIT).
- Comparison (C): Acceptable comparators included independent sedentary/non-exercise control groups, alternative exercise modalities (e.g., moderate-intensity continuous training), and within-subject pre- vs. post-exercise designs.
- Outcomes (O): At least one immunological marker measured at resting baseline (defined as samples collected <30 min prior to warm-up) and acutely post-exercise (defined as samples collected within 1 h post-intervention, with priority given to the earliest available time point (0–15 min) for the primary analysis), including: (i) immune cells: total leukocyte count, neutrophils, lymphocytes and their subsets (e.g., T/B/NK cells), and monocytes; (ii) cytokines: interleukin-6 (IL-6), interleukin-10 (IL-10), and tumor necrosis factor-alpha (TNF-α); and (iii) mucosal immunity: salivary immunoglobulin A (sIgA) and lysozyme.
- Study Design (S): Experimental studies assessing the acute effects of SIT, such as randomized controlled trials (RCTs), crossover trials, and single-group pre-post studies. Studies reporting only the effects of long-term training adaptations were explicitly excluded.

### 2.3 Information sources and search strategy

A systematic literature search was conducted in the PubMed, Web of Science, SPORTDiscus, the Cochrane Library, Embase, and Scopus electronic databases from their inception to November 2025. The search strategy combined keywords related to the intervention (“sprint interval training,” “SIT,” and “high-intensity interval training”) and the outcomes (“immune system,” “leukocyte,” “cytokine,” and “sIgA”). Additionally, the reference lists of the included studies and relevant reviews were manually screened to identify any potentially missed articles.

### 2.4 Study selection

Two independent reviewers (WL and SX) performed the study selection process. Initially, titles and abstracts were screened to exclude clearly irrelevant articles. Subsequently, the full texts of potentially eligible studies were retrieved and assessed for final inclusion. Any disagreements during the screening process were resolved through discussion and consensus or, if necessary, by consulting a third reviewer (WH).

### 2.5 Data extraction

Data were independently extracted by two reviewers (WL and YH) using a standardized data extraction form, and the results were cross-checked for accuracy. The extracted data included: (1) primary study information (authors and year of publication); (2) participant characteristics (sample size *N*, sex, age, and training status); (3) detailed SIT protocol parameters (modality, number of repetitions, sprint duration, work-to-rest ratio, and total sprint duration); (4) comparator design; and (5) statistical data for outcome measures at pre- and post-exercise time points, including mean, standard deviation (SD), and sample size (*N*).

### 2.6 Study quality

The methodological quality of the included studies was assessed by two independent reviewers using the Tool for the Assessment of Study Quality and Reporting in Exercise (TESTEX) scale. This 15-point scale is specifically designed for exercise science research and is applicable to both randomized and non-randomized study designs, ensuring a consistent assessment across all included articles in our review (13). The scoring criteria include the specification of eligibility criteria (1 point), randomization (1 point), allocation concealment (1 point), baseline group similarity (1 point), assessor blinding (1 point), follow-up rates (up to 3 points), use of intention-to-treat analysis (1 point), reporting of between-group comparisons (up to 2 points), use of point estimates and variability data (1 point), monitoring of the control group (1 point), and detailed reporting of the exercise intervention (2 points).

### 2.7 Statistical analyses

A quantitative synthesis was performed through a meta-analysis. Due to the variability in units and measurement methods for the outcomes, the standardized mean difference (SMD) was used as the primary effect measure, with Hedges’ g for small sample correction. A random-effects model was used for data pooling, given the anticipated heterogeneity in participant characteristics and SIT protocols. Inter-study heterogeneity was assessed using the Cochran’s *Q* test and quantified with the *I*^2^ statistic, with *I*^2^ > 50% indicating substantial heterogeneity. To explore the sources of this heterogeneity and to test the robustness of the findings, we conducted pre-specified subgroup analyses (based on physical activity level, SIT protocol characteristics, and sex) and leave-one-out sensitivity analyses, respectively. A formal assessment of publication bias was not performed, as fewer than 10 studies were included in any single outcome analysis. Finally, the overall certainty of the evidence for each outcome was rated using the GRADE methodology. To further explore the sources of heterogeneity and potential dose–response relationships, non-linear quadratic meta-regression analysis was performed for continuous variables (repetition duration). Additionally, subgroup analyses based on training status were conducted to address the high variability observed in cytokines and mucosal immunity. All statistical analyses were conducted in R (version 4.5.0) using the “meta” and “metafor” packages.

## 3 Results

### 3.1 Study selection

**Figure 1:**
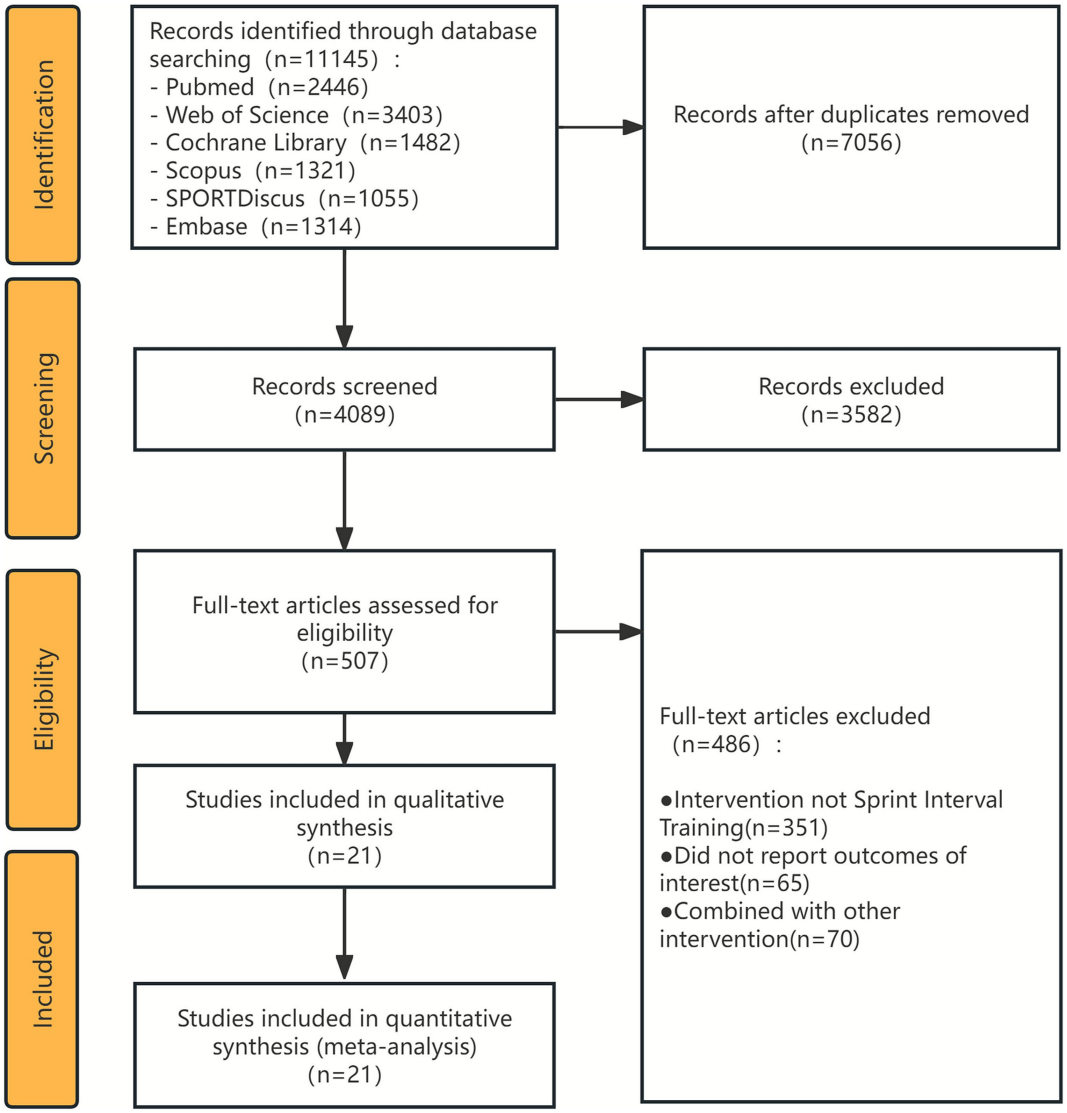
PRISMA flow diagram detailing the study selection process. The flow diagram illustrates the process of study identification, screening, eligibility assessment, and final inclusion in the systematic review and meta-analysis, following the Preferred Reporting Items for Systematic Reviews and Meta-Analyses (PRISMA) guidelines.

The systematic literature search initially yielded 11,145 records. Following the removal of 7,056 duplicates, the titles and abstracts of the remaining 4,089 articles were screened, leading to the exclusion of 3,582 records that were irrelevant to the research question. The full texts of the remaining 507 articles were then assessed for eligibility. Based on the pre-specified PICOS inclusion and exclusion criteria, a final selection of 21 studies (8, 10, 14–32) was included in this systematic review and meta-analysis. The detailed study selection process is illustrated in the PRISMA flow diagram in Figure 1.

### 3.2 Characteristics of the studies

A total of 21 studies, comprising 359 participants, were included in this meta-analysis (Table 1). The participant cohort was predominantly male, with 14 studies exclusively recruiting men, two exclusively recruiting women, and the remaining three including mixed-sex groups. The studies featured a heterogeneous population, encompassing a wide spectrum of physical activity levels from untrained adolescents and sedentary adults to recreationally active individuals and trained regional-to-professional-level athletes. The age of participants also varied considerably, ranging from adolescent cohorts with a mean age as low as 15.5 years to young adults up to 29 years old.

**Table 1:** Characteristics of the included studies.

Study	Population	Physical activity level	N	Men ratio (%)	Age (years)	Immunological outcomes
Arroyo et al. (28)	Recreationally active men	Active	11	100	23 ± 4	Lymphocyte subsets, CD69 expression, and CX3CR1 expression
Arroyo-Morales et al. (14)	Healthy active subjects	Active	50	50	22.4 ± 3.4	Salivary immunoglobulin A (sIgA)
Belviranli et al. (15)	Healthy, non-smoking, and sedentary men	Sedentary	10	100	18–24	Total white cell count and white cell subgroups
Davison (16)	Healthy, recreationally active men	Active	9	100	27 ± 5	Neutrophil oxidative burst activity, neutrophil degranulation (elastase), sIgA, and lysozyme
de Almeida-Neto et al. (17)	Male intermittent sport athletes	Trained athletes	29	100	23.2 ± 2.1	Leukocyte subsets (neutrophils, monocytes, lymphocytes, T/B cells, and NK cells) and cytokines (IL-1β, IL-6, IL-8, IL-10, IL-12p70, and TNF-α)
de Araujo et al. (18)	Professional soccer athletes	Trained athletes	32	100	21.2 ± 4.2	Salivary immunoglobulin A (Ig-A), cortisol, and total protein
Fahlman et al. (10)	Active, adult females	Active	26	0	24.2 ± 5.8	Secretory IgA (sIgA)
Ferreira et al. (19)	Physically active men	Active	20	100	25.7 ± 5.0	Plasma cytokines (IL-6, IL-10, and TNF-α) and creatine kinase (CK)
Friedman et al. (20)	Healthy subjects	Active	8	50	24 ± 2.0	Lymphocyte subsets (CD4^+^, CD8^+^, CD19^+^, etc.), apoptosis marker (annexin V), and migration marker (CX3CR1)
Goods et al. (21)	Trained male Australian footballers	Trained athletes	10	100	20.5 ± 1.9	Interleukin-6 (IL-6)
Harnish and Sabo (29)	Healthy, minimally active adults	Active	15	87	23.8 ± 3.5	Plasma cytokines (IL-6, IL-10, and TNF-α)
Jamurtas et al. (22)	Healthy young men	Active	12	100	22.4 ± 0.5	Complete blood count (white blood cells, lymphocytes, monocytes, and granulocytes)
Lee et al. (23)	Well-trained male canoe/kayak athletes	Trained athletes	22	100	15.9 ± 2.3	Leukocytes, neutrophils, lymphocytes, lymphocyte subsets, total and lipopolysaccharide (LPS)-stimulated neutrophil elastase release
Mackinnon and Jenkins (24)	Male university physical education students	Active	12	100	17–25	Salivary IgA, IgG, and IgM concentrations and flow rates
McFadden et al. (25)	Healthy, physically active adults	Active	16	50	21.2 ± 2.8	White blood cells, neutrophils, lymphocytes, monocytes, eosinophils, basophils, NK cells, B/T-lymphocytes, CD4/CD8 cells, IL-1β, IL-6, and IL-10
Meckel et al. (30)	Elite junior handball players	Trained athletes	12	100	20.3 ± 1.0	Interleukins (IL-6, IL-1β, IL-1ra, and IL-10)
Meckel et al. (31)	Elite junior handball players	Trained athletes	12	100	20.3 ± 1.0	Interleukins (IL-6, IL-1β, and IL-1ra)
O’Carroll et al. (32)	Active, healthy individuals	Active	12	67	29 ± 2	Total leukocytes, lymphocytes, T-cell subsets (CD3^+^, CD4^+^, and CD8^+^), and monocytes
Thomas et al. (26)	Apparently healthy female adolescents	Active	19	0	15.5 ± 0.6	Salivary immunoglobulin A
Verbickas et al. (27)	Healthy, physically active young men	Active	10	100	22.6 ± 5.2	Pro-inflammatory (IL-6) and anti-inflammatory (IL-10) cytokines
Wahl et al. (8)	Male triathletes/cyclists	Trained athletes	12	100	24.7 ± 3.4	Leukocytes, lymphocytes, neutrophils, mixed cell count, platelets, and derived cellular inflammation markers [neutrophil-to-lymphocyte ratio (NLR), platelet-to-lymphocyte ratio (PLR), and systemic immune-inflammation index (SII)]

This table summarizes the key characteristics of the 16 studies included in the meta-analysis, detailing the study population, physical activity level, sample size (*N*), gender distribution (men ratio %), mean age, and the specific immunological outcomes measured.

A high degree of heterogeneity was evident in the design of the sprint interval training (SIT) protocols (Table 2). The predominant exercise modality was cycle ergometry, employed in 13 studies, with running (four studies) and kayaking (one study) also represented. Repetition duration was most commonly 30 s (eight studies), with six other studies utilizing sprints of less than 10 s. The total number of repetitions ranged from as few as 3 to as many as 27. These variations resulted in a substantial range in the total sprint duration per session, from a minimum of 45 s to a maximum of 450 s. Furthermore, the prescribed work-to-rest ratios were highly variable, spanning from an intense 2:1 to a prolonged recovery ratio of 1:36. In summary, the intervention protocols demonstrated significant divergence in modality, sprint duration, total work, and recovery strategies, providing a basis for subsequent meta-regression and subgroup analyses to explore sources of heterogeneity.

**Table 2:** Details of the exercise intervention protocols.

Study	Group	Exercise modality	Session duration (min)	Intensity	*N* reps	Rep duration (s)	Work-to-rest ratio	Total sprint duration (s)
Arroyo et al. (28)	SIT	Cycling	53	130% Wmax	15	20	1:8	300
	MICT	Cycling	53	65% VO_2_max	—	—	—	—
Arroyo-Morales et al. (14)	SIT	Cycling	~9	All-out (Wingate)	3	30	1:6	90
Belviranli et al. (15)	SIT	Cycling	~16	All-out (Wingate)	4	30	1:8	120
Davison (16)	SIT	Cycling	~16	All-out (Wingate)	4	30	1:8	120
	Control	Resting	—	—	—	—	—	—
de Almeida-Neto et al. (17)	SIT	Running	~19	Maximal sprints	18 (3 × 6)	35 m	N/A	~90
de Araujo et al. (18)	SIT	Running	~4.5	Maximal sprints	7	~6–7	1:4	45
Fahlman et al. (10)	SIT	Cycling	~8	All-out (Wingate)	3	30	1:6	90
Ferreira et al. (19)	SIT	Cycling	~10	All-out sprints	13	30	2:1	390
Friedman et al. (20)	SIT	Cycling	~14	All-out (Wingate)	6 (2 × 3)	30	1:4	180
Goods et al. (21)	SIT	Cycling	~16	Maximal sprints	27 (3 × 9)	5	1:4	135
Harnish and Sabo (29)	SIT (Wingate)	Cycling	~25	All-out	5	30	1:9	150
	SIT (Tabata)	Cycling	~15	All-out (~170% VO_2_max)	10	20	2:1	200
Jamurtas et al. (22)	SIT	Cycling	~14	All-out sprints	4	30	1:8	120
	MICT	Cycling	30	70% VO_2_max	—	—	—	—
Lee et al. (23)	SIT	Kayak	~30	Maximum intensity paddling	6	90	1:2.7	450
Mackinnon and Jenkins (24)	SIT	Cycling	~30	Supramaximal exercise	5	60	1:5	300
McFadden et al. (25)	SIT	Cycling	~22	All-out (WAnT)	9 (1 + 8)	30 & 10	01:12	120
Meckel et al. (30)	SIT	Running	~12	Fixed-pace supramaximal SIT	4	250 m	~1:4	<180
Meckel et al. (31)	SIT	Running	~12	Fixed-pace supramaximal SIT	4	100–400 m	Variable	<180
O’Carroll et al. (32)	SIT	Cycling	~20	Maximal	6	20	1:6	120
	MICT	Cycling	45	70% VO_2_peak	—	—	—	—
Thomas et al. (26)	SIT	Cycling	~4	Maximal sprints	6	8	1:3.75	48
Verbickas et al. (27)	SIT	Cycling	~38	All-out cycling bouts	12	5	01:36	60
Wahl et al. (8)	SIT	Cycling	~10	All-out (Wingate)	4	30	01:15	120

This table provides a detailed breakdown of the sprint interval training (SIT) protocols used in each included study. For studies that included comparator groups, details of the moderate-intensity continuous training (MICT) or non-exercise control conditions are also provided. Parameters such as exercise modality, number of repetitions (*N* reps), duration of each repetition, and the work-to-rest ratio are presented. N/A (not applicable) is used for parameters related to chronic training interventions.

The immunological outcomes measured across the studies were extensive and can be broadly categorized into three domains: (1) Circulating immune cell counts: This included absolute counts and percentages of total leukocytes, neutrophils, lymphocytes, and monocytes. A subset of studies also provided a more granular analysis of lymphocyte subpopulations (e.g., CD4^+^, CD8^+^, B cells, and NK cells) and functional neutrophil metrics. (2) Plasma cytokine concentrations: This category encompassed key pro- and anti-inflammatory cytokines, with interleukin-6 (IL-6) and interleukin-10 (IL-10) being the most frequently investigated, alongside others such as tumor necrosis factor-alpha (TNF-α). (3) Mucosal immunity markers: This domain primarily focused on the concentration and flow rate of salivary immunoglobulin A (sIgA), with a few studies also assessing other markers such as salivary lysozyme.

### 3.3 Methodological quality assessment

The methodological quality of the 21 included studies was assessed using the TESTEX scale. The total scores ranged from 6 to 12 out of a possible 15. Based on the predefined criteria, seven studies were classified as high quality, seven as moderate quality, and seven as low quality. Common methodological weaknesses included lack of randomization (D1) in single-group pre-post designs and lack of assessor blinding (D5). The quality classification for each study is presented in Table 3.

**Table 3:** Consolidated TESTEX scoring results.

Study	1	2	3	4	5	Study quality score (0–5)	6	7	8	9	10	11	12	13	14	15	Study reporting score (0–10)	Total (0–15)	Study quality classification
Arroyo et al. (28)	1	1	0	1	0	3	0	0	1	1	0	1	1	1	1	—	6	9	Moderate quality
Arroyo-Morales et al. (14)	1	0	0	—	0	1	1	0	0	1	—	1	1	0	1	1	6	7	Moderate quality
Belviranli et al. (15)	1	0	0	—	0	1	1	0	0	1	—	1	1	0	1	—	5	6	Low quality
Davison (16)	1	1	0	1	1	4	1	0	1	1	1	1	1	0	1	—	7	11	High quality
de Almeida-Neto et al. (17)	1	0	0	1	0	2	1	0	1	1	—	1	1	0	1	—	6	8	Moderate quality
de Araujo et al. (18)	1	0	0	—	0	1	1	0	0	1	—	1	1	0	1	—	5	6	Low quality
Fahlman et al. (10)	1	0	0	—	0	1	1	0	0	1	—	1	1	0	1	0	5	6	Low quality
Ferreira et al. (19)	1	1	0	1	1	4	1	0	1	1	—	1	1	0	1	—	6	10	High quality
Friedman et al. (20)	1	0	0	—	0	1	1	0	0	1	—	1	1	0	1	0	5	6	Low quality
Goods et al. (21)	1	1	1	1	1	5	1	0	1	1	—	1	1	0	1	—	6	11	High quality
Harnish and Sabo (29)	1	1	0	1	0	3	1	1	1	1	1	1	1	0	1	1	9	12	High quality
Jamurtas et al. (22)	1	1	0	1	0	3	1	0	1	1	—	1	1	0	1	—	6	9	Moderate quality
Lee et al. (23)	1	0	0	—	0	1	1	0	0	1	—	1	1	0	1	—	5	6	Low quality
Mackinnon and Jenkins (24)	1	0	0	—	0	1	1	0	0	1	—	1	1	0	1	—	5	6	Low quality
McFadden et al. (25)	1	1	1	1	1	5	1	0	1	1	—	1	1	0	1	0	6	11	High quality
Meckel et al. (30)	1	0	0	—	1	2	1	1	0	1	0	1	1	0	1	—	6	8	Moderate quality
Meckel et al. (31)	1	1	0	1	1	4	1	0	1	1	0	1	1	0	1	—	6	10	High quality
O’Carroll et al. (32)	1	1	0	1	0	3	1	0	1	1	1	1	1	0	1	0	7	10	High quality
Thomas et al. (26)	1	0	0	—	0	1	1	0	0	1	—	1	1	0	1	0	5	6	Low quality
Verbickas et al. (27)	1	1	0	1	0	3	1	0	1	1	—	1	1	0	1	—	6	9	Moderate quality
Wahl et al. (8)	1	1	0	1	0	3	1	0	1	1	—	1	1	0	1	—	6	9	Moderate quality

“—” indicates not applicable. TESTEX criteria key: (1) Eligibility criteria specified? (2) Random allocation? (3) Allocation concealed? (4) Groups similar at baseline? (5) Assessor blinding? (6) >85% follow-up? (7) Intention-to-treat analysis? (8) Between-group comparison reported? (9) Point measure and variability reported? (10) Activity monitoring in the control group? (11) Relative exercise intensity monitored? (12) Exercise volume and adherence reported? (13) Adverse events reported? (14) Activity level of subjects specified? (15) Cycling of female participants controlled?

### 3.4 Meta-analysis, subgroup analysis, and meta-regression

**Figure 2:**
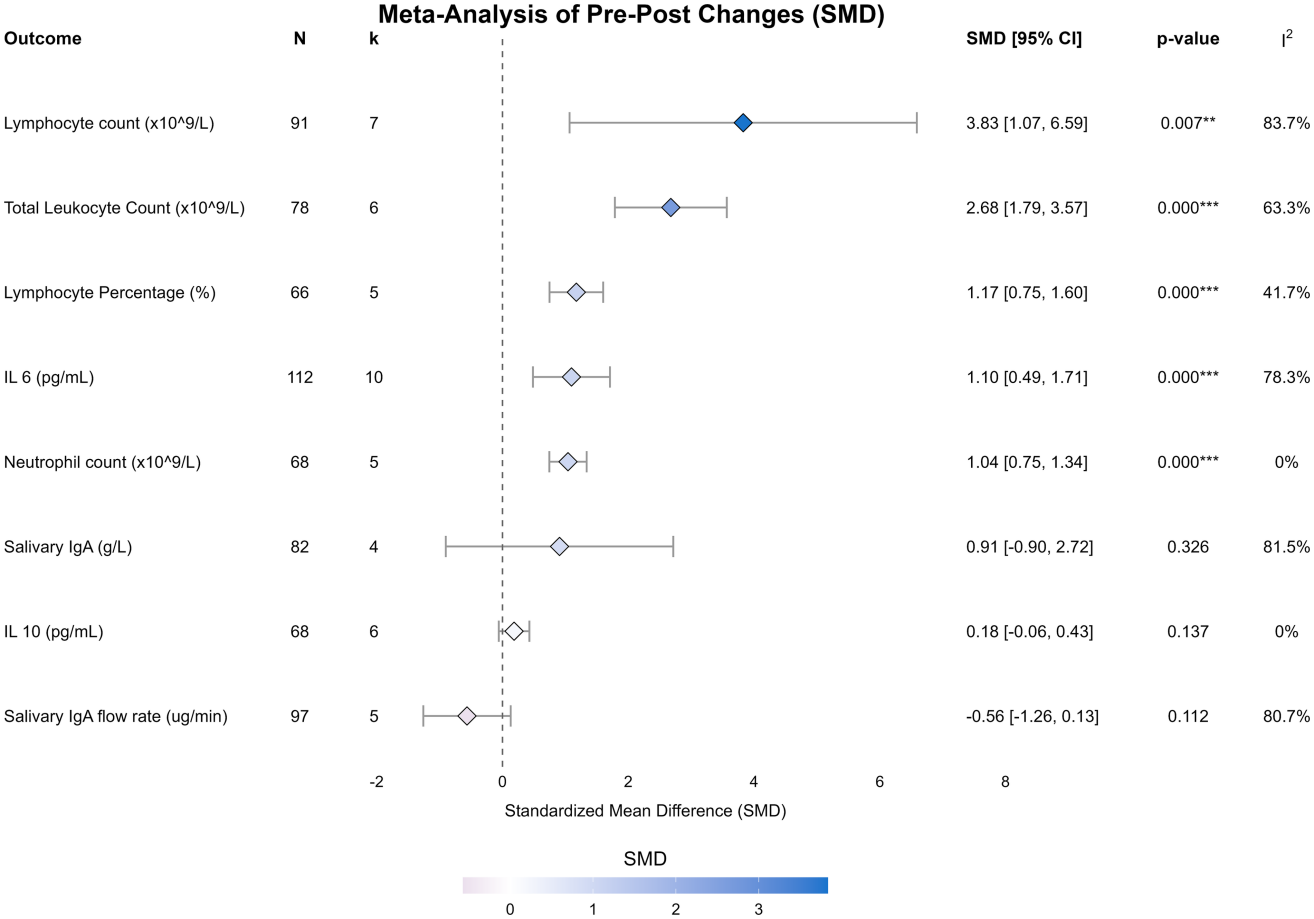
Forest plots of the effect of sprint interval training on immunological outcomes. Forest plots display the standardized mean difference (SMD) and 95% confidence intervals (CI) for the acute effect of a single session of sprint interval training on total leukocyte count, neutrophil count, lymphocyte count, lymphocyte percentage, interleukin-6, interleukin-10, salivary IgA flow rate, and salivary IgA concentration. The size of the squares represents the weight of each study, and the diamond represents the pooled effect estimate from the random-effects model.

**Figure 3:**
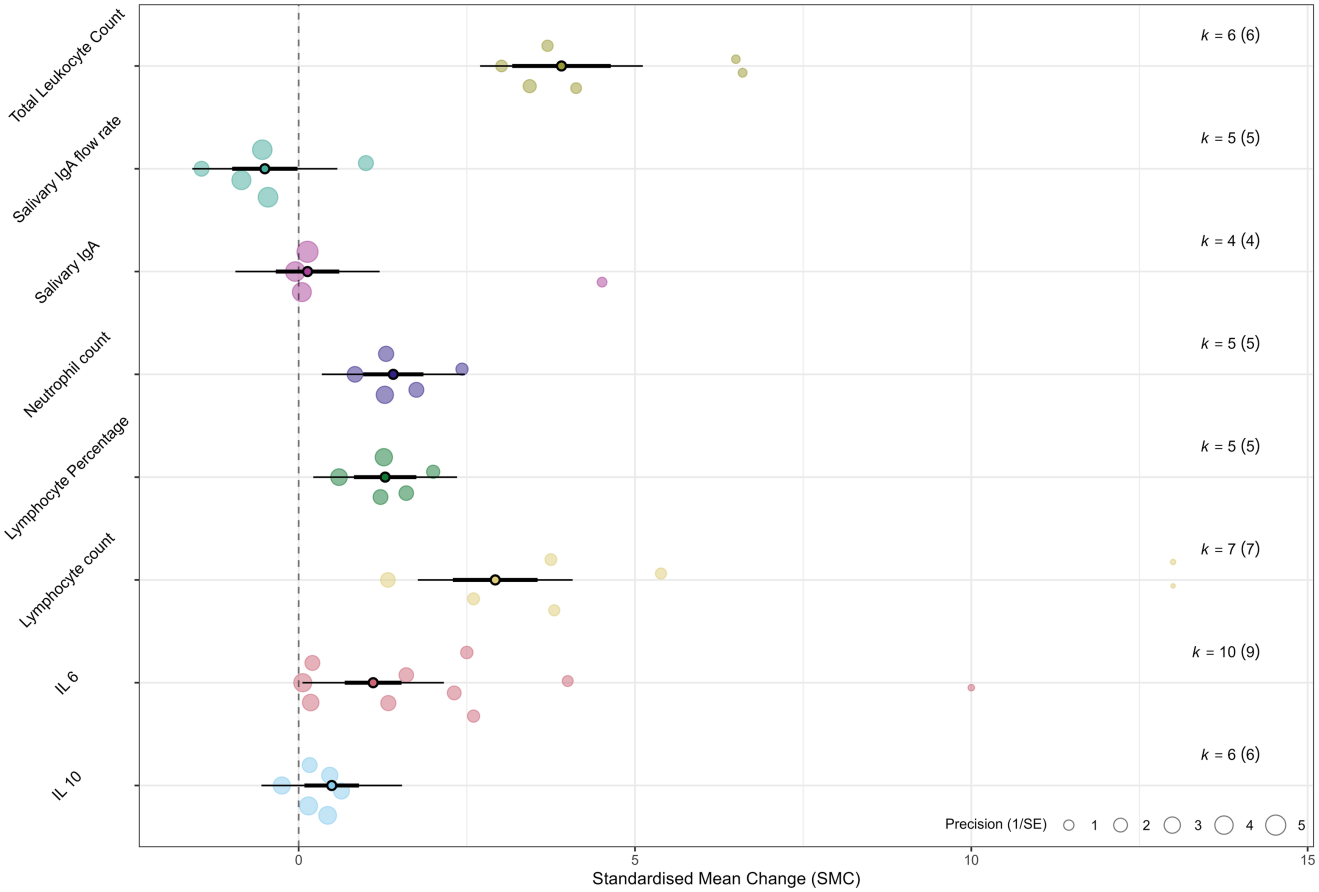
Orchard plot summarizing the overall effects. An orchard plot visualizes the pooled effect estimates (SMD) and 95% confidence intervals for all analyzed immunological outcomes. Each point represents the overall SMD for an outcome, with the horizontal lines indicating the 95% CI. The plot provides a comprehensive overview of the magnitude and direction of the acute immune response to sprint interval training.

The summary of pooled effect sizes is presented in Table 4, with individual forest plots in Figure 2 and an overall orchard plot in Figure 3. To address the high heterogeneity observed in the primary analysis, detailed results from the pre-specified subgroup analyses and meta-regressions are presented below.

**Table 4:** Summary of meta-analysis results for each outcome.

Outcome	No. studies	*N*	Pooled SMD (95% CI)	*p*	Heterogeneity *I*^2^, *p*
IL 10 (pg/mL)	6	68	0.18 [−0.06, 0.43]	0.137	0%, 0.52
IL 6 (pg/mL)	10	112	1.10 [0.49, 1.71]	0.000^**^	78.3%, < 0.001
Lymphocyte count (×10^9^/L)	7	91	3.83 [1.07, 6.59]	0.007^**^	83.7%, < 0.001
Lymphocyte percentage (%)	5	66	1.17 [0.75, 1.60]	0.000^**^	41.7%, 0.14
Neutrophil count (×10^9^/L)	5	68	1.04 [0.75, 1.34]	0.000^**^	0%, 0.84
Salivary IgA (g/L)	4	82	0.91 [−0.90, 2.72]	0.326	81.5%, 0.00
Salivary IgA flow rate (μg/min)	5	97	−0.56 [−1.26, 0.13]	0.112	80.7%, < 0.001
Total leukocyte count (×10^9^/L)	6	78	2.68 [1.79, 3.57]	0.000^**^	63.3%, 0.02

Data include the number of studies (No. studies) and total participants (*N*) for each outcome, the pooled standardized mean difference (SMD) with its 95% confidence interval (95% CI), and measures of statistical heterogeneity (*I*^2^ and its corresponding *p*-value). ^*^p < 0.05 and ^**^p < 0.01.

#### 3.4.1 Acute effects on plasma cytokine concentrations

**Figure 4:**
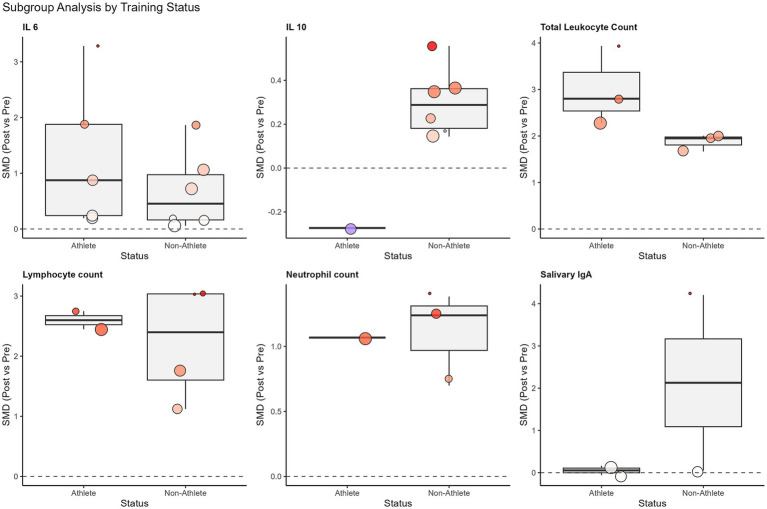
Subgroup analysis of standardized mean differences (SMD) for immunological outcomes based on participant training status (athletes vs. non-athletes). The box plots display the distribution of effect sizes for each subgroup, with individual study estimates represented by colored dots (sized by weight). The thick horizontal line within the box represents the median effect size. The distinct stability of salivary IgA was noted in athletes compared to the variability in non-athletes, and the robust elevation of IL-6 was noted in both groups.

A single session of SIT induced a large and significant elevation in plasma interleukin-6 (IL-6) [10 studies, *N* = 112; SMD = 1.10, 95% CI (0.49, 1.71), *p* < 0.001]. Although heterogeneity was high (*I*^2^ = 78.3%), the subgroup analysis revealed that training status acted as a critical moderator (Figure 4). Specifically, trained athletes exhibited a twofold greater IL-6 response compared to untrained individuals (SMD: 1.20 vs. 0.63), suggesting an enhanced metabolic myokine release in adapted muscle.

Regarding protocol density, a potential inverted U-shaped trend was observed for the work-to-rest ratio and IL-6 response (Supplementary Figure S1). This trend suggests that moderate ratios (approximately 1:1 to 1:5) might maximize the IL-6 response compared to extreme ratios, although this relationship did not reach statistical significance. In contrast to IL-6, the anti-inflammatory cytokine interleukin-10 (IL-10) showed only a small, non-significant increase (SMD = 0.18, *p* = 0.137).

#### 3.4.2 Acute effects on circulating immune cell counts

SIT elicited a robust and uniform mobilization of innate immune cells. Total leukocyte count increased substantially (SMD = 2.68, *p* < 0.001). Notably, neutrophil count demonstrated a large, significant increase (SMD = 1.04, *p* < 0.001) with zero heterogeneity (*I*^2^ = 0%), indicating a highly conserved physiological stress response independent of protocol variations. Lymphocyte count (SMD = 3.83) also increased significantly.

**Figure 5:**
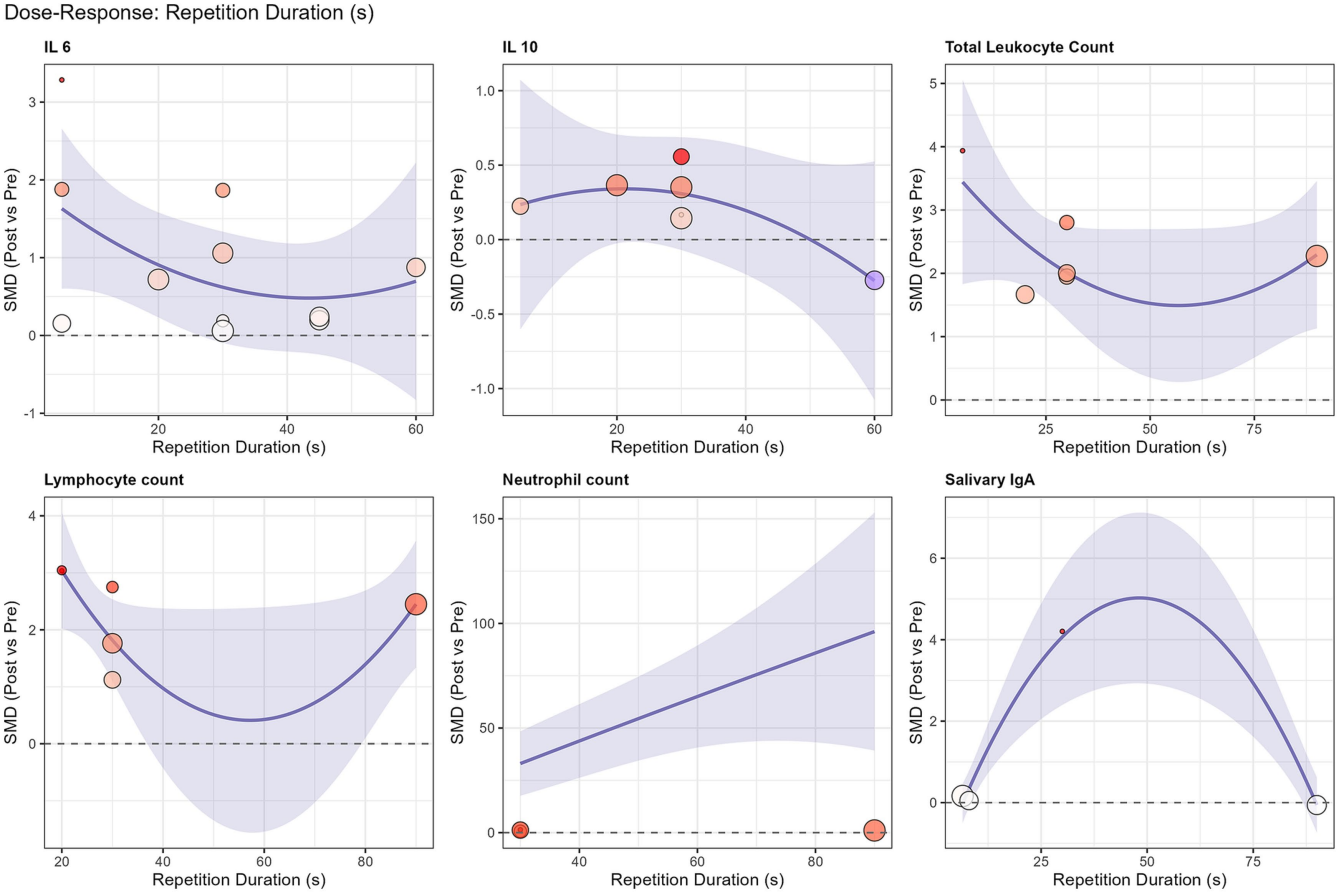
Non-linear quadratic meta-regression analysis of repetition duration (s) on the standardized mean difference (SMD) for immunological outcomes. The solid blue line represents the predicted effect size, and the shaded area indicates the 95% confidence interval. A U-shaped dose–response relationship is observed for lymphocyte count (*R*^2^ = 48.6%), suggesting greater mobilization with very short (<10 s) and prolonged (>45 s) sprint efforts.

Exploratory non-linear meta-regression analysis revealed a U-shaped dose–response relationship for lymphocyte count (*R*^2^ = 48.6%). As illustrated in Figure 5, both very short (<10 s) and prolonged (>45 s) sprint repetitions appeared to elicit stronger lymphocyte mobilization compared to moderate-duration sprints. However, no significant dose–response relationship was found for total session sprint duration regarding systemic immune responses (Supplementary Figure S2), indicating that the supramaximal intensity, rather than total volume, is the primary driver of cellular mobilization.

#### 3.4.3 Acute effects on mucosal immunity markers

The overall analysis for salivary IgA (sIgA) flow rate (SMD = −0.56) and concentration (SMD = 0.91) revealed no statistically significant acute effects, accompanied by high heterogeneity (*I*^2^ > 80%). However, subgroup analysis based on training status clarified this variability (Figure 4). A striking divergence was observed: while untrained individuals showed erratic responses with a trend toward immunosuppression, trained athletes maintained remarkably stable sIgA levels post-exercise [SMD = 0.07, 95% CI (−0.31, 0.45)] with zero heterogeneity (*I*^2^ = 0%). This finding indicates that SIT does not compromise mucosal barrier function in trained populations.

### 3.5 Sensitivity and robustness analysis

Leave-one-out sensitivity analyses were performed to assess the influence of individual studies on the pooled effect estimates (Supplementary Figures S5–S13). The robust increases observed for total leukocyte count, neutrophil count, and IL-6 were not driven by any single study, as the pooled effect sizes remained significant and stable across all iterations. Specifically for IL-6, despite high heterogeneity, the direction and magnitude of the effect were consistent. For lymphocyte count, while the effect remained significant, the variation in effect magnitude upon exclusion of certain studies [e.g., Wahl et al. (8)] mirrored the high heterogeneity observed in the main analysis. For IL-10 and sIgA measures, the non-significant overall findings were stable, with no single study omission altering the statistical conclusion, further supporting the influence of moderating factors such as training status.

### 3.6 Publication Bias

The potential for publication bias was considered. However, in line with methodological recommendations, formal testing for funnel plot asymmetry (e.g., via Egger’s test) was not performed, as the number of studies included in any single outcome analysis was fewer than 10, limiting the power and reliability of such tests.

### 3.7 Summary of evidence

The overall certainty of the evidence for each outcome was assessed using the GRADE framework (Table 5). The evidence supporting the acute mobilization of innate immune cells was rated as moderate certainty. Specifically, total leukocyte count, neutrophil count, and lymphocyte percentage received this rating, primarily driven by the large magnitude of the observed effects. Notably, the certainty for neutrophil count was further bolstered by the complete absence of statistical heterogeneity (*I*^2^ = 0%). Regarding systemic inflammation, the evidence for the elevation of IL-6 was rated as low certainty; although the effect size was large, the rating was downgraded due to serious inconsistency (*I*^2^ = 78.3%) and risk of bias inherent in the primary studies. Finally, evidence for salivary IgA outcomes was rated as very low certainty due to serious inconsistency and imprecision in the pooled analysis, although this variability was largely mitigated within the trained athlete subgroup.

**Table 5:** GRADE summary of findings.

Outcome	Studies (*k*)/participants (N)	Effect estimate [SMD (95% CI)]	Certainty of evidence (GRADE)	Justification for rating
Total leukocyte count	6/78	2.68 [1.79, 3.57] (very large increase)	⚫⚫⚫⚪ Moderate	Downgraded once for risk of bias^a^; upgraded once for very large magnitude of effect^c^
Neutrophil count	5/68	1.04 [0.75, 1.34] (large increase)	⚫⚫⚫⚪ Moderate	Downgraded once for risk of bias^a^; upgraded once for large magnitude of effect^c^. Heterogeneity is very low (*I*^2^ = 0%)
Lymphocyte count	7/91	3.83 [1.07, 6.59] (very large increase)	⚫⚫⚪⚪ Low	Downgraded once for risk of bias^a^ and once for serious inconsistency^b^ (*I*^2^ = 83.7%). Magnitude is very large, but heterogeneity precludes upgrading
Lymphocyte percentage	5/66	1.17 [0.75, 1.60] (large increase)	⚫⚫⚫⚪ Moderate	Downgraded once for risk of bias^a^; upgraded once for large magnitude of effect^c^. Inconsistency is not serious (*I*^2^ = 41.7%)
Interleukin-6 (IL-6)	10/112	1.10 [0.49, 1.71] (large increase)	⚫⚫⚪⚪ Low	Downgraded once for risk of bias^a^ and once for serious inconsistency^b^ (*I*^2^ = 78.3%). Imprecision downgrade removed as CI does not cross zero
Interleukin-10 (IL-10)	6/68	0.18 [−0.06, 0.43] (no significant difference)	⚫⚫⚪⚪ Low	Downgraded once for risk of bias^a^ and once for imprecision^d^ (CI crosses zero). Heterogeneity is low (*I*^2^ = 0%)
Salivary IgA flow rate	5/97	−0.56 [−1.26, 0.13] (no significant difference)	⚫⚪⚪⚪ Very low	Downgraded once for risk of bias^a^, once for serious inconsistency^b^ (*I*^2^ = 80.7%), and once for imprecision^d^
Salivary IgA concentration	4/82	0.91 [−0.90, 2.72] (no significant difference)	⚫⚪⚪⚪ Very low	Downgraded once for risk of bias^a^, once for serious inconsistency^c^ (*I*^2^ = 81.5%), and once for imprecision^d^

a Risk of bias: Downgraded due to serious risk of bias, as the body of evidence includes studies with high or some concerns for bias.

b Inconsistency: Downgraded due to serious inconsistency, as heterogeneity was high (*I*^2^ > 75%) and unexplained.

c Large effect: Upgraded because the magnitude of the effect was large (SMD >0.8), and the confidence interval was precise.

d Imprecision: Downgraded due to serious imprecision, as the 95% confidence interval was wide and crossed the line of no effect.

## 4 Discussion

This systematic review and meta-analysis provides the first quantitative synthesis of the acute immunological responses to a single session of sprint interval training (SIT). The main findings of this study are summarized in the graphical abstract. Our core findings indicate that SIT is a potent physiological stressor that elicits a rapid, large-scale mobilization of circulating immune cells. This response is characterized by a highly conserved immediate post-exercise increase in total leukocytes and neutrophils and a dose-dependent mobilization of lymphocytes. Concurrently, SIT orchestrates a complex cytokine milieu, characterized by a significant pro-inflammatory/myokine signal (IL-6) that is markedly enhanced in trained individuals, alongside a smaller, non-significant trend for the anti-inflammatory cytokine IL-10. Furthermore, our subgroup analysis revealed that mucosal immunity (sIgA) remains stable in trained athletes, suggesting a specific adaptation to high-intensity stress.

### 4.1 Profound immune cell mobilization: conserved response and dose-dependency

The most robust finding of this study is the profound exercise-induced leukocytosis following SIT. Notably, the neutrophil response exhibited zero heterogeneity (*I*^2^ = 0%), indicating that this rapid demargination is a fundamental, highly conserved physiological stress response to SIT that operates independently of specific protocol variations or participant characteristics. This phenomenon is primarily attributed to the catecholamine surge and increased hemodynamic shear stress during supramaximal exercise, leading to the demargination of leukocytes from the vascular endothelium and storage pools (33).

Regarding lymphocytes, our meta-regression analysis revealed a U-shaped dose–response relationship, suggesting that both extremely short (<10 s) and prolonged (>45 s) sprint repetitions elicit stronger mobilization than intermediate durations. This likely reflects a biphasic stimulus: short sprints rely on rapid neural activation and mechanical shear, while prolonged efforts drive mobilization via metabolic accumulation (e.g., acidosis). This finding challenges a simplistic interpretation of the traditional “open-window” theory. The modern perspective suggests that this transient lymphocytosis represents a functional redeployment of cells to peripheral tissues to enhance local surveillance, rather than a state of systemic immune compromise (2).

### 4.2 The complexity of the cytokine response: training status as a key modulator

In contrast to the relatively uniform response of immune cells, the pattern of cytokine alterations reveals a more nuanced regulatory network. While our updated analysis confirmed a significant overall elevation in IL-6, the subgroup analysis provided a critical explanation for the high heterogeneity observed in previous literature: training status is a core determinant. SIT induced a twofold greater IL-6 response in trained athletes compared to untrained individuals. This supports the “myokine hypothesis,” where muscle-derived IL-6 acts as an energy sensor to promote substrate availability during high metabolic demand, rather than reflecting pathological inflammation (34, 35).

Conversely, the anti-inflammatory cytokine IL-10 showed only a small, non-significant increasing trend (*p* = 0.137) in the pooled analysis. This suggests that while an anti-inflammatory counter-regulation exists, it may be less consistent acutely, or its peak may occur outside the immediate sampling window used in many studies (36).

### 4.3 Immune resilience in the mucosal immune response

Regarding salivary IgA (sIgA), the first line of defense against upper respiratory tract infections, our initial analysis showed high heterogeneity. However, stratification by training status revealed a striking divergence. While untrained individuals showed erratic responses, trained athletes exhibited remarkably stable sIgA levels post-exercise (*I*^2^ = 0%). This finding indicates that chronic adaptations to training confer a degree of “immune resilience,” allowing athletes to tolerate the stress of supramaximal SIT without compromising their mucosal barrier function. Therefore, fears of SIT-induced mucosal suppression appear unwarranted for adapted populations.

### 4.4 Strengths, limitations, and future directions

A primary strength of this study is the use of meta-regression and subgroup analyses to dissect sources of heterogeneity, identifying training status and protocol dose as key moderators. Nevertheless, several limitations should be acknowledged. First, the included studies exhibit significant heterogeneity in their SIT protocols and participant characteristics. Although partially explained through subgroup analysis, this remains a major challenge in the field. Second, acute immune responses are highly time-dependent. Although we prioritized “immediately post-exercise” data, slight variations in sampling timing (e.g., 0 vs. 15 min post) across included studies likely contribute to residual heterogeneity, particularly for cytokines and sIgA. Finally, the majority of participants were male, limiting generalizability to females.

Future research should focus on the standardized reporting of SIT protocols and include more female and diverse age-group participants. There is a need for studies investigating the immune dynamics over a longer recovery period (>24 h) and exploring the link between these acute responses and long-term training adaptations and infection risk. Employing a broader range of immunological metrics, including functional assays, will help to create a more comprehensive picture of the immunological landscape following SIT.

## 5 Conclusion

This systematic review and meta-analysis provides quantitative evidence indicating that a single session of sprint interval training (SIT) elicits a rapid and potent acute response from the immune system in healthy individuals. The hallmark of this response is a significant and highly conserved mobilization of circulating total leukocytes, neutrophils, and lymphocytes, indicating an immediate, systemic cellular immune activation in response to high-intensity exercise stress. Concurrently, SIT orchestrates a complex cytokine milieu, characterized by a significant pro-inflammatory/myokine signal (IL-6) that is strongly modulated by the individual’s training background, alongside a smaller, non-significant trend for IL-10. Regarding mucosal immunity, our analysis revealed that sIgA levels remain stable in trained athletes, contrasting with the variability seen in untrained individuals. In conclusion, SIT acts as a powerful immunological stressor; however, its response pattern—particularly in trained individuals—may reflect a functional, adaptive process rather than a state of simple immunosuppression. These findings underscore the importance of considering an individual’s training status when applying SIT to optimize training loads and recovery strategies.

## Data Availability

The original contributions presented in the study are included in the article/Supplementary material, further inquiries can be directed to the corresponding author.
