# Supplementary material for: The acute effect of sprint interval training on the immune system: a systematic review and meta-regression analysis
Source: Front Med (Lausanne). 2026 Feb 2;12:1703829. doi: 10.3389/fmed.2025.1703829 (PMC12908032; doi:10.3389/fmed.2025.1703829)

# Supplemental Online Content

Table S1. Search Strategy.

Table S2. Summary of subgroup analyses for outcomes.

Figure S1. Dose-response meta-regression of Work:Rest Ratio on immunological outcomes.

Figure S2. Dose-response meta-regression of Total Sprint Duration (s) on immunological outcomes.

Figure S3. Risk of bias summary.

Figure S4. Risk of bias traffic light plot.

Figures S5-S13. Leave-one-out sensitivity analyses.

# Table S1. Search Strategy.

Pubmed

| #1 | "sprint interval training"[Title/Abstract] OR "sprint interval exercise"[Title/Abstract] OR "sprint intermittent training"[Title/Abstract] OR "sprint training"[Title/Abstract] OR "sprint-interval training"[Title/Abstract] OR "High-Intensity Interval Training"[Mesh] OR "High intensity intermittent training"[Title/Abstract] OR "High-intensity intermittent training"[Title/Abstract] OR "Interval training"[Title/Abstract] OR "interval exercise"[Title/Abstract] OR "HIIT"[Title/Abstract] OR "high intensity exercise"[Title/Abstract] OR "high intensity aerobic interval training"[Title/Abstract] OR "all-out exercise training"[Title/Abstract] OR "all-out training"[Title/Abstract] OR "all-out interval training"[Title/Abstract] OR "Supramaximal interval training"[Title/Abstract] OR "Wingate training"[Title/Abstract] OR "maximal intensity"[Title/Abstract] |
| --- | --- |
| #2 | "Immune System"[Mesh] OR "Immunity"[Mesh] OR "Leukocytes"[Mesh] OR "Lymphocytes"[Mesh] OR "Neutrophils"[Mesh] OR "Monocytes"[Mesh] OR "Killer Cells, Natural"[Mesh] OR "Cytokines"[Mesh] OR "Interleukins"[Mesh] OR "Tumor Necrosis Factor-alpha"[Mesh] OR "Immunoglobulins"[Mesh] OR "Inflammation"[Mesh]) OR immune*[Title/Abstract] OR immunity[Title/Abstract] OR immunoglobulin*[Title/Abstract] OR IgA[Title/Abstract] OR leukocyte*[Title/Abstract] OR lymphocyte*[Title/Abstract] OR "white blood cell*"[Title/Abstract] OR "T-Cell*"[Title/Abstract] OR "B-Cell*"[Title/Abstract] OR "NK cell*"[Title/Abstract] OR "natural killer"[Title/Abstract] OR neutrophil*[Title/Abstract] OR monocyte*[Title/Abstract] OR cytokine*[Title/Abstract] OR interleukin*[Title/Abstract] OR IL-6[Title/Abstract] OR IL-10[Title/Abstract] OR TNF[Title/Abstract] OR "tumor necrosis factor"[Title/Abstract] OR inflammat*[Title/Abstract] OR immunomodulat*[Title/Abstract] |
| #3 | #1 AND #2 |

Web of Science

| #1 | TS=("sprint interval training" OR "sprint interval exercise" OR "Sprint intermittent training" OR "sprint training" OR "sprint-interval training" OR "High-Intensity Interval Training" OR "High intensity intermittent training" OR "High-intensity intermittent training" OR "Interval training" OR "interval exercise" OR "HIIT" OR "high intensity exercise" OR "high intensity aerobic interval training" OR "all-out exercise training" OR "all-out training" OR "all-out interval training" OR "Supramaximal interval training" OR "Wingate training" OR "maximal intensity") |
| --- | --- |
| #2 | TS=(immune* OR immunity OR immunoglobulin* OR IgA OR leukocyte* OR lymphocyte* OR "white blood cell*" OR "T-Cell*" OR "B-Cell*" OR "NK cell*" OR "natural killer" OR neutrophil* OR monocyte* OR cytokine* OR interleukin* OR IL-6 OR IL-10 OR TNF OR "tumor necrosis factor" OR inflammat* OR immunomodulat*) |
| #3 | #1 AND #2 |

SPORTDiscus

| S1 | TI "sprint interval training" OR AB "sprint interval training" OR TI "sprint interval exercise" OR AB "sprint interval exercise" OR TI "supramaximal interval training" OR AB "supramaximal interval training" OR TI "supramaximal interval exercise" OR AB "supramaximal interval exercise" OR TI "all-out interval training" OR AB "all-out interval training" OR TI "repeated sprint training" OR AB "repeated sprint training" OR TI "repeated sprint exercise" OR AB "repeated sprint exercise" OR TI "high-intensity interval training" OR AB "high-intensity interval training" OR TI HIIT OR AB HIIT) OR (DE "HIGH intensity interval training") ) AND (DE "EXERCISE" OR DE "SPORTS" OR DE "PHYSICAL activity") |
| --- | --- |
| S2 | DE "Immunity" OR DE "Leukocytes" OR DE "Lymphocytes" OR DE "Neutrophils" OR DE "Cytokines" OR DE "Immunoglobulins" OR DE "Inflammation") OR TI ( immune* OR immunity OR immunoglobulin* OR IgA OR leukocyte* OR lymphocyte* OR "white blood cell*" OR cytokine* OR interleukin* OR inflammat* ) OR AB ( immune* OR immunity OR immunoglobulin* OR IgA OR leukocyte* OR lymphocyte* OR "white blood cell*" OR cytokine* OR interleukin* OR inflammat* |
| S3 | S1 AND S2 |

Cochrane Library

| #1 | (sprint interval training):ti,ab,kw OR (sprint interval exercise):ti,ab,kw OR (supramaximal interval training):ti,ab,kw OR (supramaximal interval exercise):ti,ab,kw OR (all-out interval training):ti,ab,kw OR (repeated sprint training):ti,ab,kw OR (repeated sprint exercise):ti,ab,kw OR (high-intensity interval training):ti,ab,kw OR (HIIT):ti,ab,kw |
| --- | --- |
| #2 | [mh Exercise] OR [mh "Exercise Therapy"] OR [mh "Physical Education and Training"] OR [mh Sports] |
| #3 | #1 AND #2 |
| #4 | MeSH descriptor: [Immune System] explode all trees |
| #5 | MeSH descriptor: [Leukocytes] explode all trees |
| #6 | MeSH descriptor: [Cytokines] explode all trees |
| #7 | immune*:ti,ab,kw OR immunity:ti,ab,kw OR immunoglobulin*:ti,ab,kw OR leukocyte*:ti,ab,kw OR lymphocyte*:ti,ab,kw OR neutrophil*:ti,ab,kw OR cytokine*:ti,ab,kw OR inflammat*:ti,ab,kw |
| #8 | #4 OR #5 OR #6 OR #7 |

Embase

| #1 | 'sprint interval training':ab,ti OR 'sprint interval exercise':ab,ti OR 'supramaximal interval training':ab,ti OR 'supramaximal interval exercise':ab,ti OR 'all-out interval training':ab,ti OR 'repeated sprint training':ab,ti OR 'repeated sprint exercise':ab,ti OR 'high intensity interval training'/exp OR 'high-intensity interval training':ab,ti OR 'hiit':ab,ti) AND ('exercise'/exp OR 'kinesiotherapy'/exp OR 'sport'/exp |
| --- | --- |
| #2 | 'immune system'/exp OR 'leukocyte'/exp OR 'lymphocyte'/exp OR 'neutrophil'/exp OR 'cytokine'/exp OR 'inflammation'/exp OR 'immunoglobulin'/exp |
| #3 | (immune* or immunity or immunoglobulin* or iga or leukocyte* or lymphocyte* or 'white blood cell*' or cytokine* or interleukin* or inflammat* or immunomodulat*).ti,ab,kw. |
| #4 | #2 OR #3 |
| #5 | #1 AND #4 |

Scopus

|  | TITLE-ABS-KEY("sprint interval training" OR "sprint interval exercise" OR "supramaximal interval training" OR "supramaximal interval exercise" OR "all-out interval training" OR "repeated sprint training" OR "repeated sprint exercise" OR "high-intensity interval training" OR HIIT) AND TITLE-ABS-KEY(exercise OR sport OR "physical activity") |
| --- | --- |
| AND |  |
|  | TITLE-ABS-KEY(immune* OR immunity OR immunoglobulin* OR IgA OR leukocyte* OR lymphocyte* OR "white blood cell*" OR "T-Cell*" OR "B-Cell*" OR "NK cell*" OR "natural killer" OR neutrophil* OR monocyte* OR cytokine* OR interleukin* OR IL-6 OR IL-10 OR TNF OR "tumor necrosis factor" OR inflammat* OR immunomodulat*) |

# Table S2. Summary of subgroup analyses for outcomes.

| **Outcomes** | **Subgroups** | **No.Studies** | **N** | **SMD (95%CI)** | **p** | **I²** | **p_het** |
| --- | --- | --- | --- | --- | --- | --- | --- |
| **IL 10** | **Sex** |  |  |  |  |  |  |
|  | Male | 4 | 38 | 0.14 (-0.31, 0.60) | 0.54 | 0.00% | 0.598 |
|  | Mix | 2 | 30 | 0.25 (-0.26, 0.76) | 0.341 | 0.00% | 0.689 |
|  | Test for subgroup differences: |  |  |  | 0.763 |  |  |
|  | **Rep duration** |  |  |  |  |  |  |
|  | >=30s | 4 | 43 | 0.20 (-0.23, 0.62) | 0.369 | 0.00% | 0.55 |
|  | <30s | 2 | 25 | 0.18 (-0.38, 0.73) | 0.535 | 0.00% | 0.89 |
|  | Test for subgroup differences: |  |  |  | 0.955 |  |  |
| **IL 6** | **Physical Activity Level** |  |  |  |  |  |  |
|  | Untrained | 5 | 56 | 0.63 (-0.01, 1.28) | 0.055 | 61.05% | 0.036* |
|  | Trained Athletes | 5 | 56 | 1.20 (0.14, 2.25) | 0.027* | 79.31% | < 0.001*** |
|  | Test for subgroup differences: |  |  |  | 0.375 |  |  |
|  | **Sex** |  |  |  |  |  |  |
|  | Male | 8 | 82 | 1.00 (0.28, 1.73) | 0.007** | 73.43% | < 0.001*** |
|  | Mix | 2 | 30 | 0.55 (-0.43, 1.53) | 0.273 | 71.22% | 0.062 |
|  | Test for subgroup differences: |  |  |  | 0.467 |  |  |
|  | **Rep duration** |  |  |  |  |  |  |
|  | >=30s | 6 | 67 | 0.71 (0.23, 1.19) | 0.004** | 44.34% | 0.11 |
|  | <30s | 4 | 45 | 1.26 (-0.20, 2.72) | 0.092 | 86.08% | < 0.001*** |
|  | Test for subgroup differences: |  |  |  | 0.484 |  |  |
| **Lymphocyte count** | **Physical Activity Level** |  |  |  |  |  |  |
|  | Untrained | 4 | 48 | 2.16 (1.23, 3.09) | < 0.001*** | 65.70% | 0.033* |
|  | Trained Athletes | 2 | 34 | 2.54 (1.89, 3.20) | < 0.001*** | 0.00% | 0.676 |
|  | Test for subgroup differences: |  |  |  | 0.506 |  |  |
|  | **Sex** |  |  |  |  |  |  |
|  | Male | 4 | 55 | 2.28 (1.45, 3.11) | < 0.001*** | 60.98% | 0.053 |
|  | Mix | 2 | 27 | 2.32 (1.08, 3.57) | < 0.001*** | 64.20% | 0.095 |
|  | Test for subgroup differences: |  |  |  | 0.958 |  |  |
|  | **Rep duration** |  |  |  |  |  |  |
|  | >=30s | 3 | 44 | 2.09 (1.13, 3.05) | < 0.001*** | 66.32% | 0.051 |
|  | <30s | 3 | 38 | 2.51 (1.59, 3.43) | < 0.001*** | 50.96% | 0.13 |
|  | Test for subgroup differences: |  |  |  | 0.537 |  |  |
| **Lymphocyte Percentage** | **Physical Activity Level** |  |  |  |  |  |  |
|  | Untrained | 2 | 22 | 0.78 (0.15, 1.40) | 0.014* | 0.00% | 0.352 |
|  | Trained Athletes | 3 | 44 | 1.39 (0.91, 1.86) | < 0.001*** | 0.00% | 0.547 |
|  | Test for subgroup differences: |  |  |  | 0.124 |  |  |
|  | **Sex** |  |  |  |  |  |  |
|  | Male | 5 | 66 | 1.16 (0.79, 1.54) | < 0.001*** | 9.90% | 0.35 |
| **Salivary IgA** | **Physical Activity Level** |  |  |  |  |  |  |
|  | Untrained | 2 | 28 | 2.04 (-2.03, 6.11) | 0.326 | 94.51% | < 0.001*** |
|  | Trained Athletes | 2 | 54 | 0.07 (-0.31, 0.45) | 0.707 | 0.00% | 0.571 |
|  | Test for subgroup differences: |  |  |  | 0.345 |  |  |
|  | **Rep duration** |  |  |  |  |  |  |
|  | >=30s | 2 | 31 | 1.98 (-2.19, 6.16) | 0.351 | 94.87% | < 0.001*** |
|  | <30s | 2 | 51 | 0.12 (-0.27, 0.51) | 0.542 | 0.00% | 0.784 |
|  | Test for subgroup differences: |  |  |  | 0.384 |  |  |
| **Salivary IgA flow rate** | **Physical Activity Level** |  |  |  |  |  |  |
|  | Untrained | 5 | 97 | -0.59 (-1.23, 0.06) | 0.075 | 70.03% | 0.010** |
|  | **Sex** |  |  |  |  |  |  |
|  | Mix | 2 | 50 | -0.54 (-0.94, -0.14) | 0.008** | 0.00% | 0.889 |
|  | Male | 2 | 21 | -0.41 (-2.71, 1.89) | 0.729 | 91.45% | < 0.001*** |
|  | Female | 1 | 26 | -0.94 (-1.52, -0.37) | 0.001** | N/A | N/A |
|  | Test for subgroup differences: |  |  |  | 0.515 |  |  |
|  | **Rep duration** |  |  |  |  |  |  |
|  | >=30s | 5 | 97 | -0.59 (-1.23, 0.06) | 0.075 | 70.03% | 0.010** |
| **Total Leukocyte Count** | **Physical Activity Level** |  |  |  |  |  |  |
|  | Untrained | 3 | 34 | 1.86 (1.27, 2.45) | < 0.001*** | 0.00% | 0.876 |
|  | Trained Athletes | 3 | 44 | 2.78 (1.94, 3.62) | < 0.001*** | 41.52% | 0.181 |
|  | Test for subgroup differences: |  |  |  | 0.079 |  |  |
|  | **Rep duration** |  |  |  |  |  |  |
|  | >=30s | 4 | 56 | 2.24 (1.75, 2.73) | < 0.001*** | 0.00% | 0.715 |
|  | <30s | 2 | 22 | 2.70 (0.49, 4.92) | 0.017* | 82.33% | 0.017* |
|  | Test for subgroup differences: |  |  |  | 0.688 |  |  |


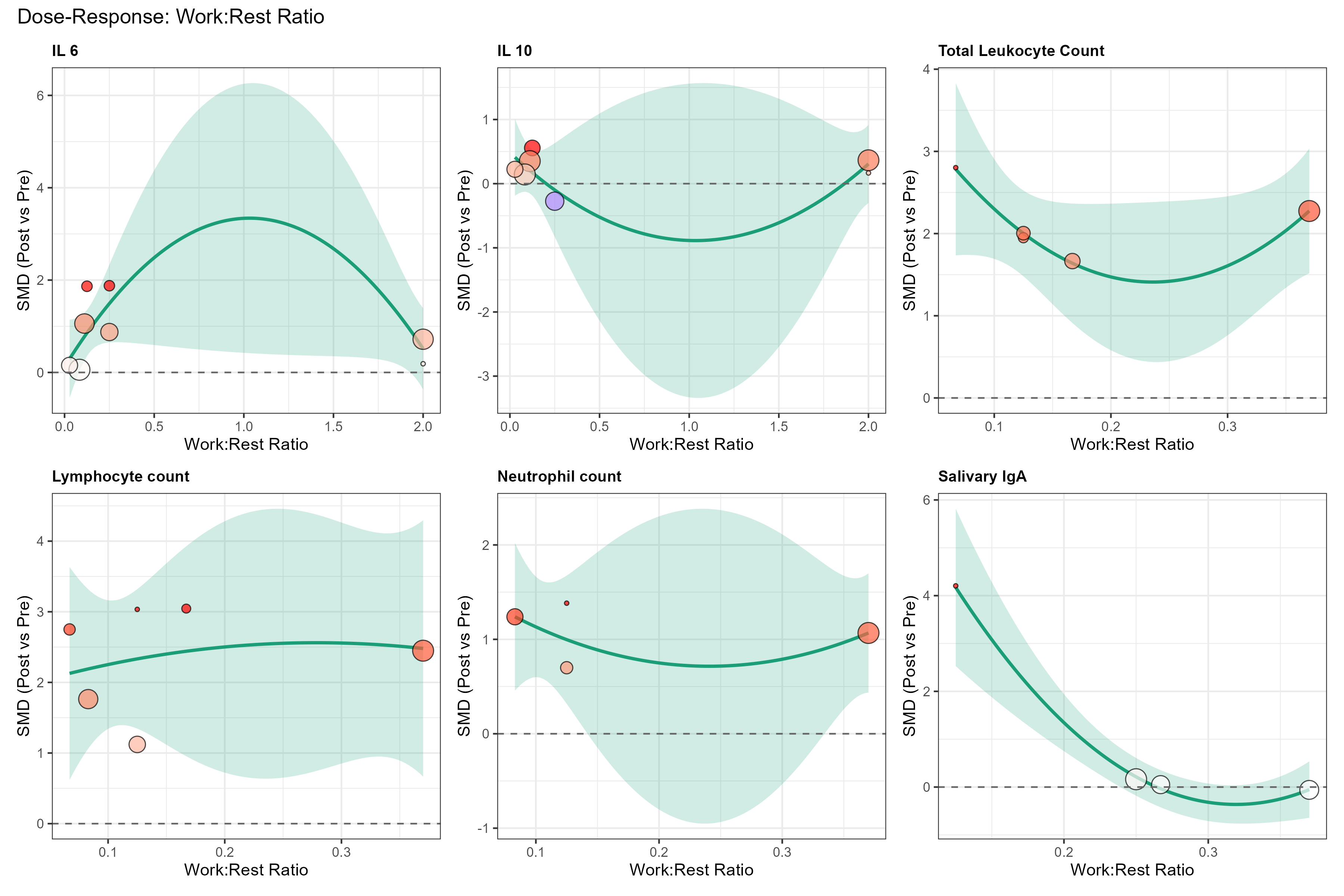


# Figure S1. Dose-response meta-regression of Work:Rest Ratio on immunological outcomes. The solid green line represents the predicted effect size with 95% CI (shaded). An inverted U-shaped trend was observed for IL-6, suggesting moderate work:rest ratios may maximize the cytokine response, though this did not reach statistical significance.


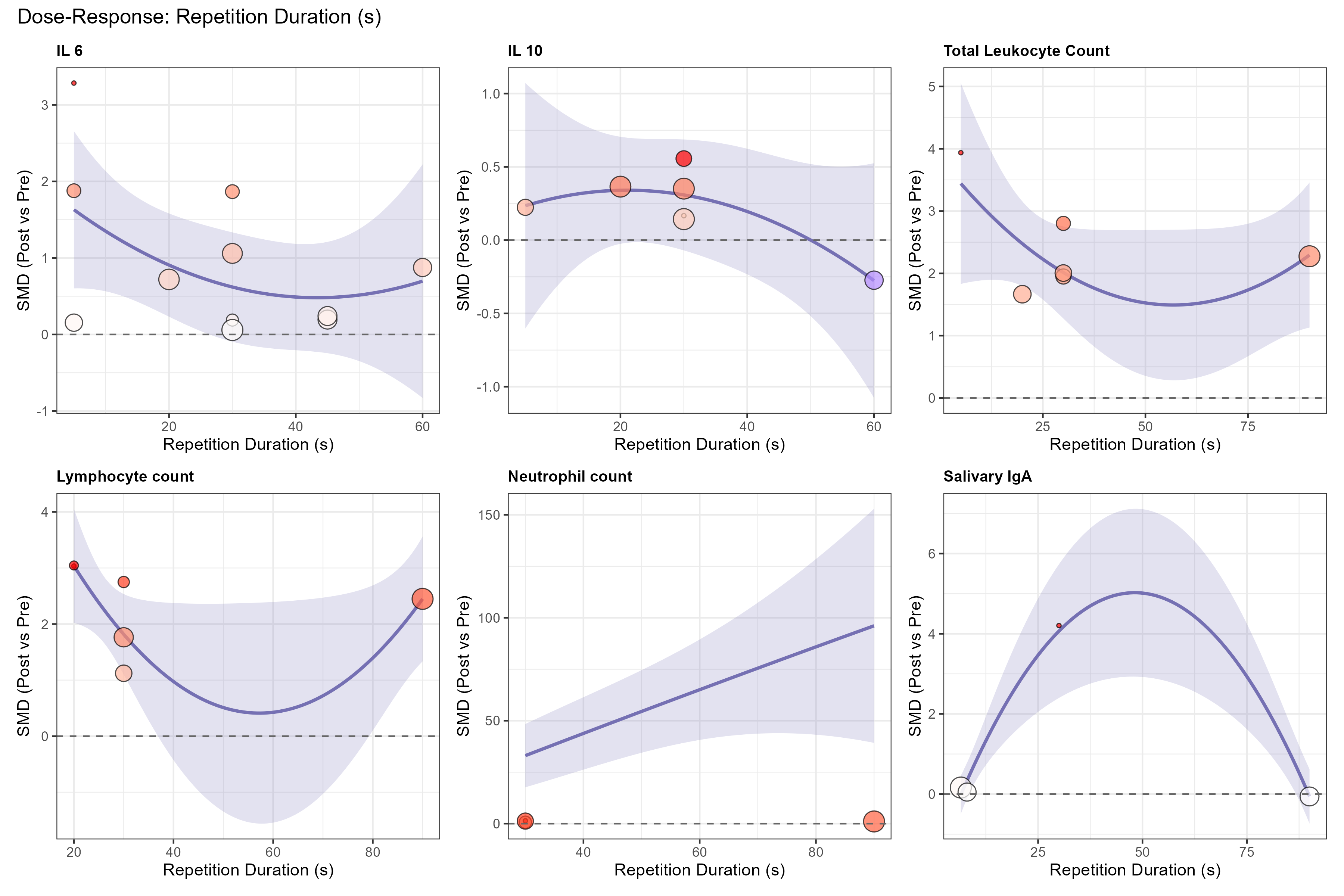


# Figure S2. Dose-response meta-regression of Total Sprint Duration (s) on immunological outcomes. The solid orange line represents the predicted effect size with 95% CI (shaded). No significant relationships were found (R2 ≈ 0%), suggesting that total volume is not the primary driver of the acute immune response.


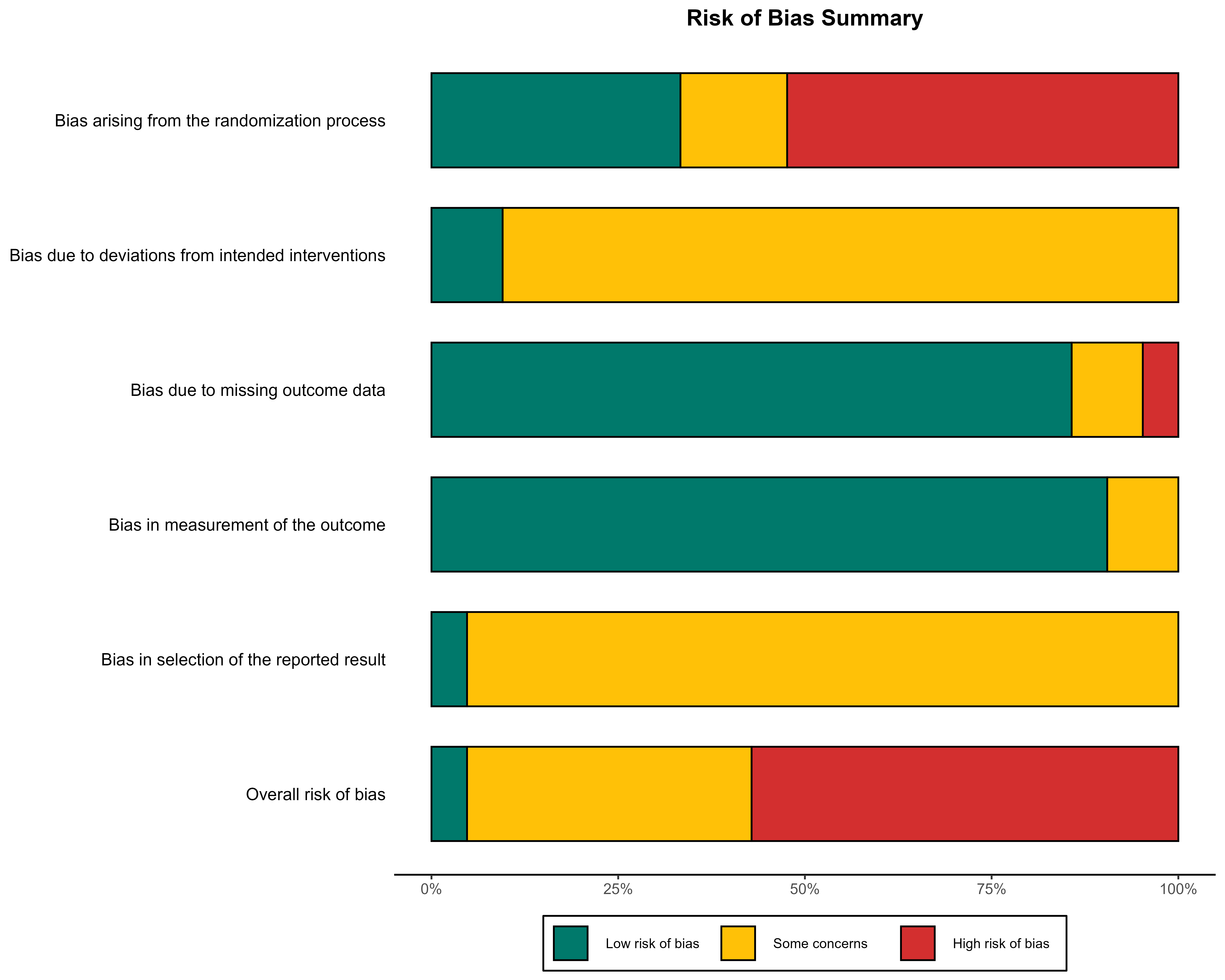


# **Figure S3. Risk of bias summary.** The bar chart displays the distribution of risk of bias judgments (low risk, some concerns, high risk) for each domain of the Cochrane RoB 2 tool across all included studies.


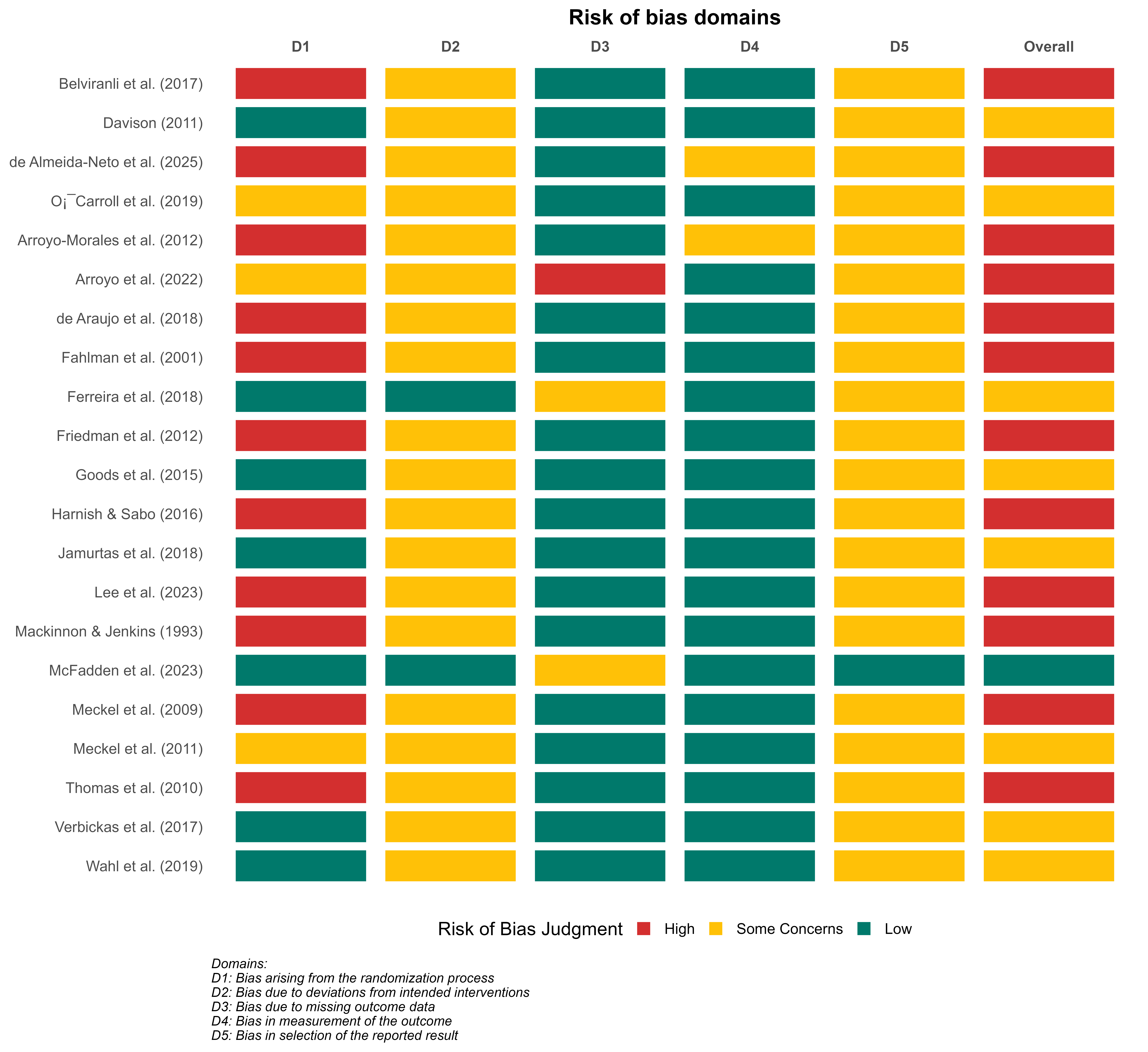


# **Figure S4. Risk of bias traffic light plot.** Each row represents an individual study, and each column represents a specific risk of bias domain from the Cochrane RoB 2 tool. Green, yellow, and red indicate low risk, some concerns, and high risk of bias, respectively.

# **Figures S5-S13. Leave-one-out sensitivity analyses.** Forest plots from the leave-one-out sensitivity analysis for each primary outcome (e.g., Total Leukocyte Count, IL-6, etc.). Each row represents the pooled effect estimate calculated after removing the specified study, demonstrating the influence of individual studies on the overall result. The vertical line indicates the original pooled effect estimate including all studies.


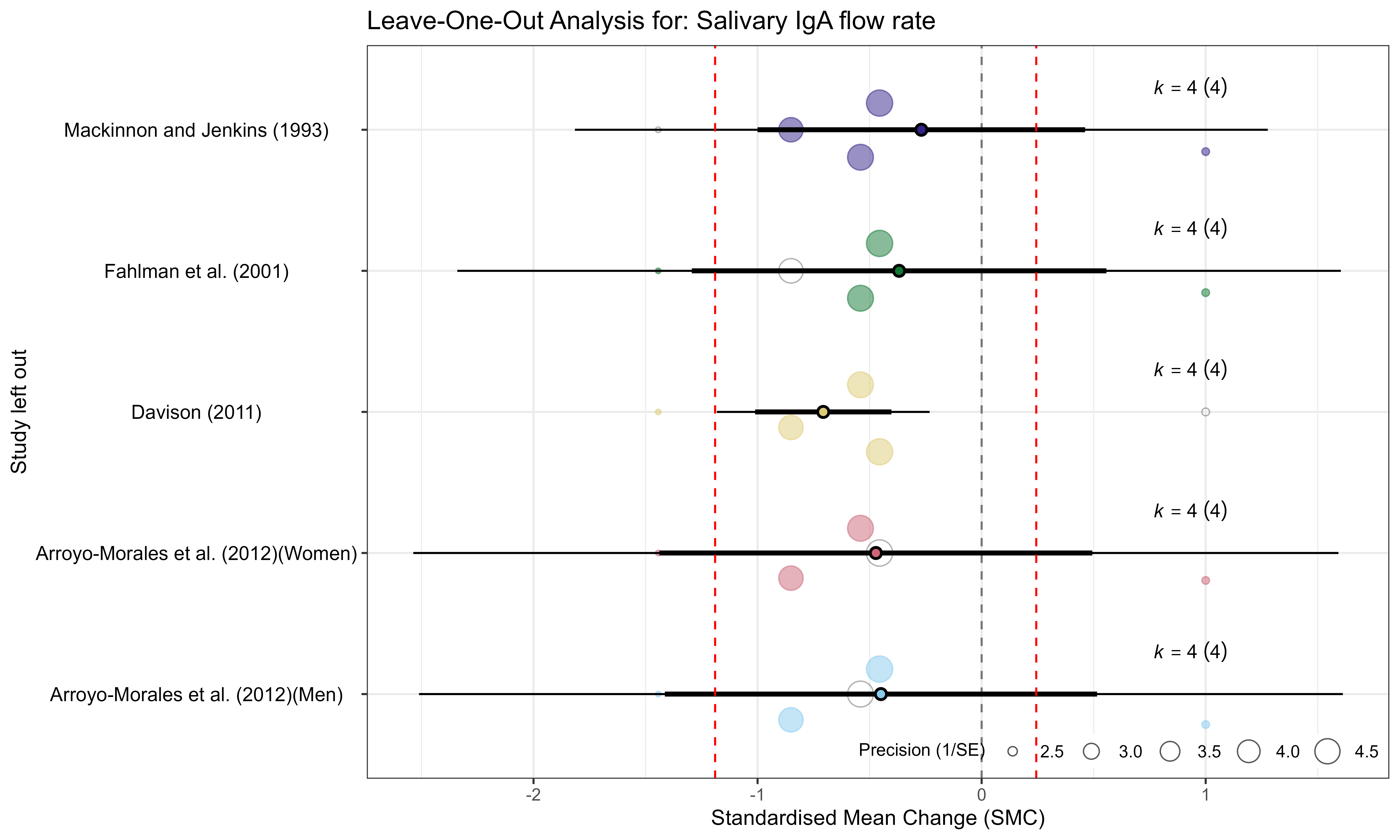

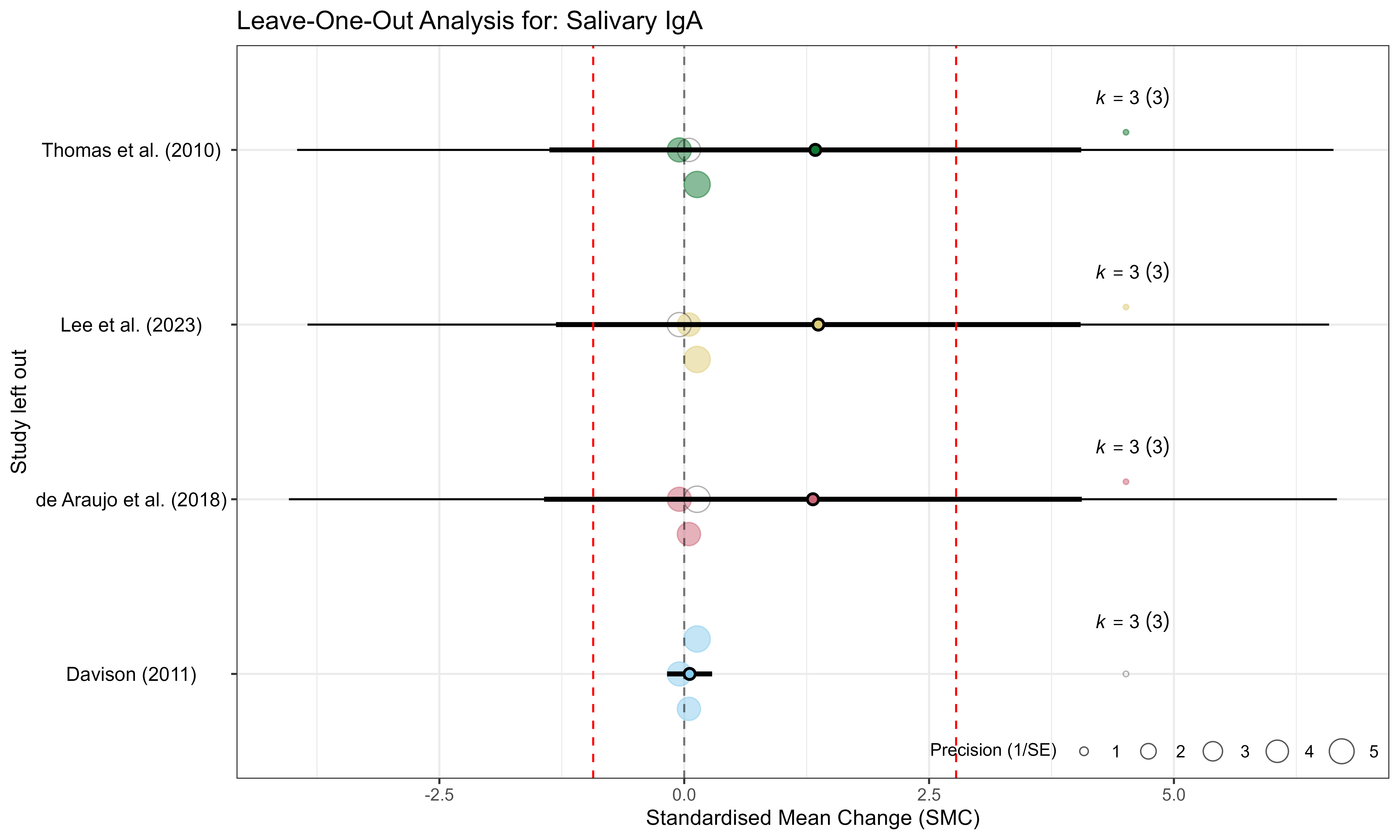

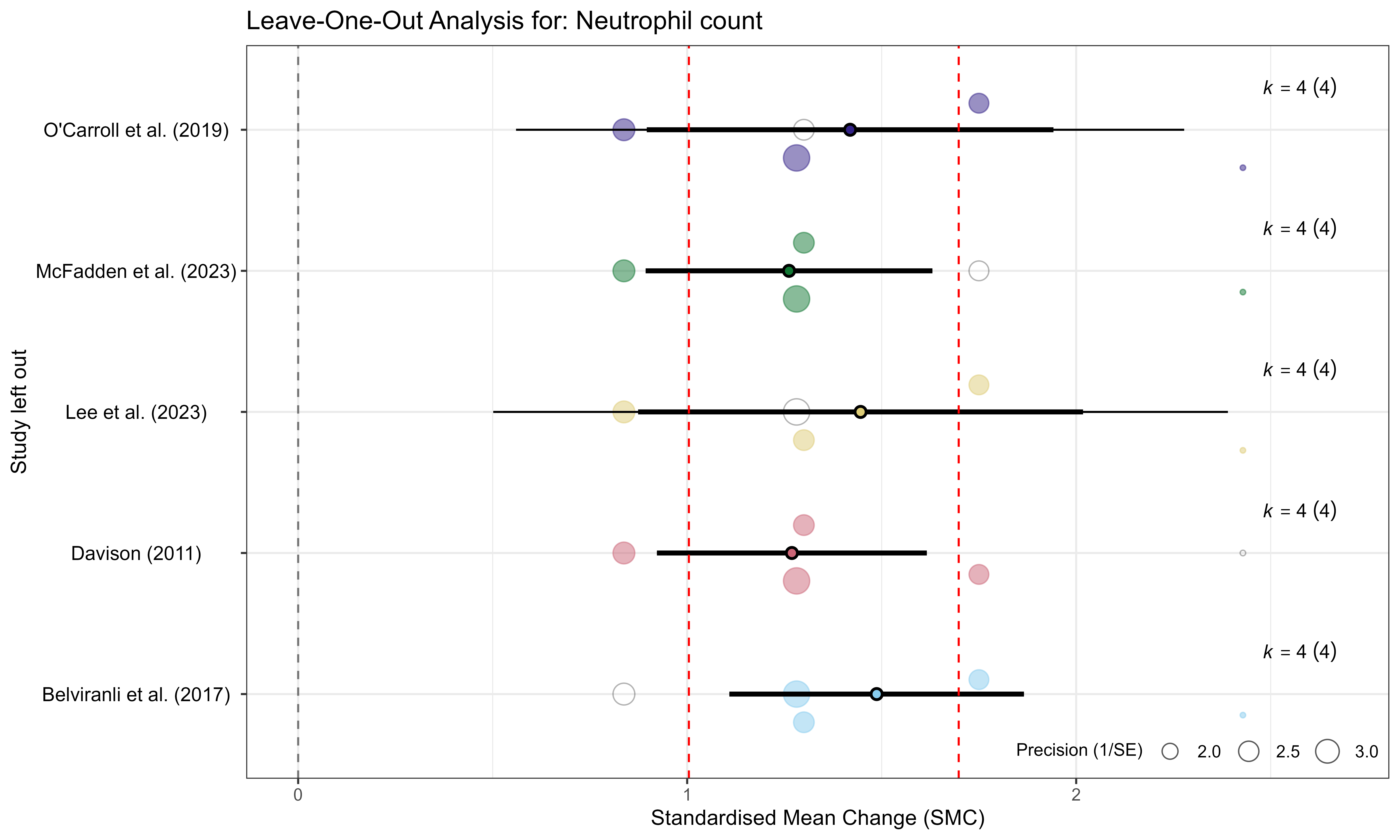

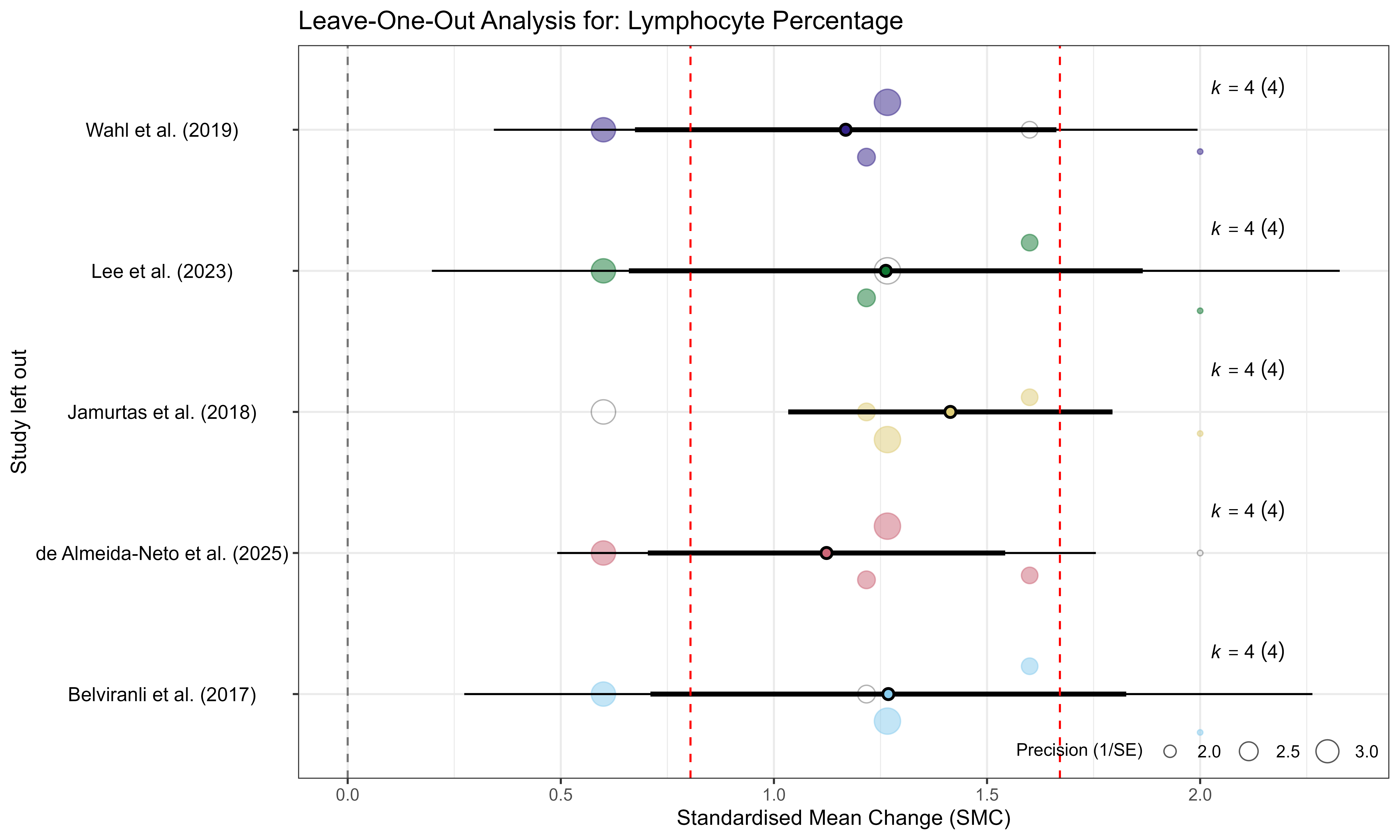

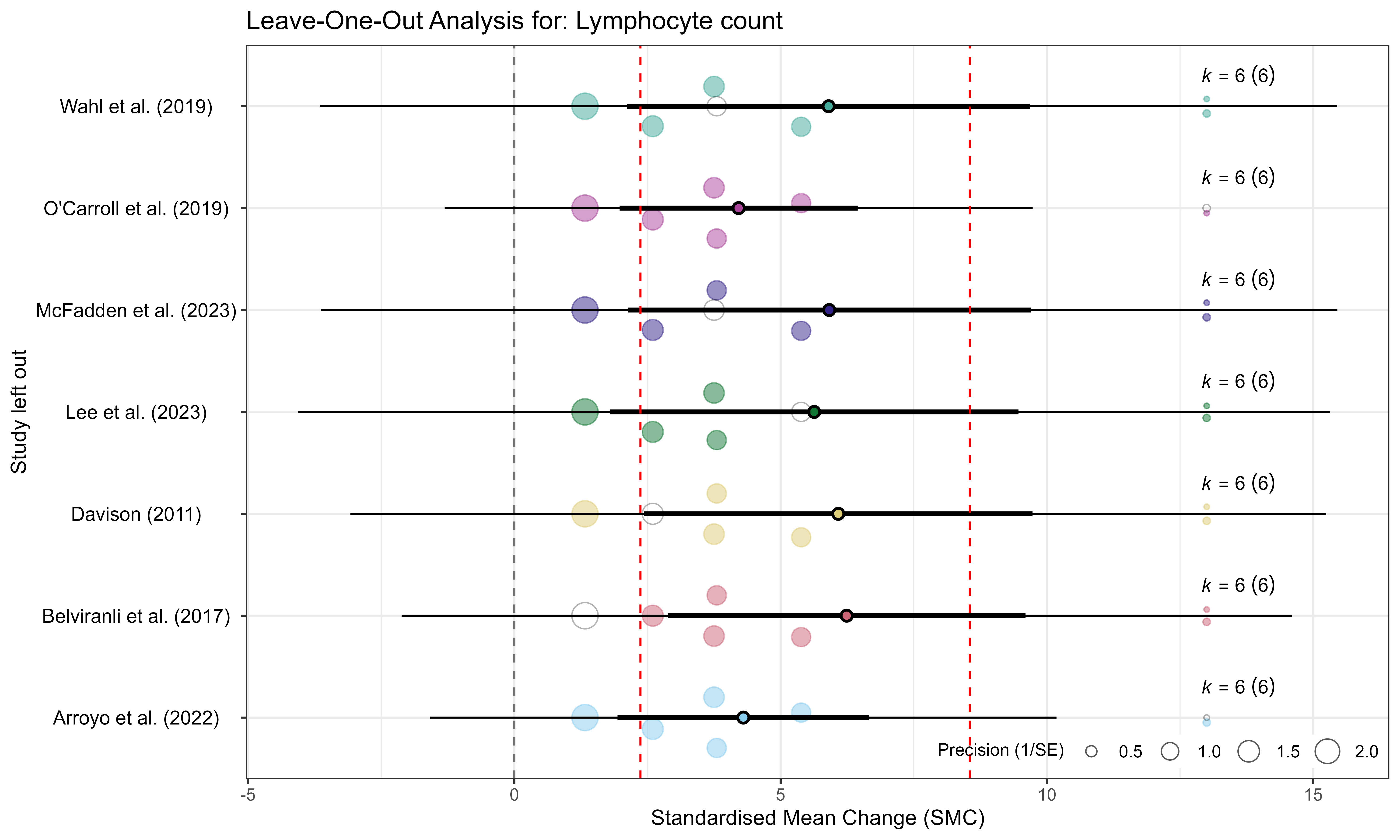

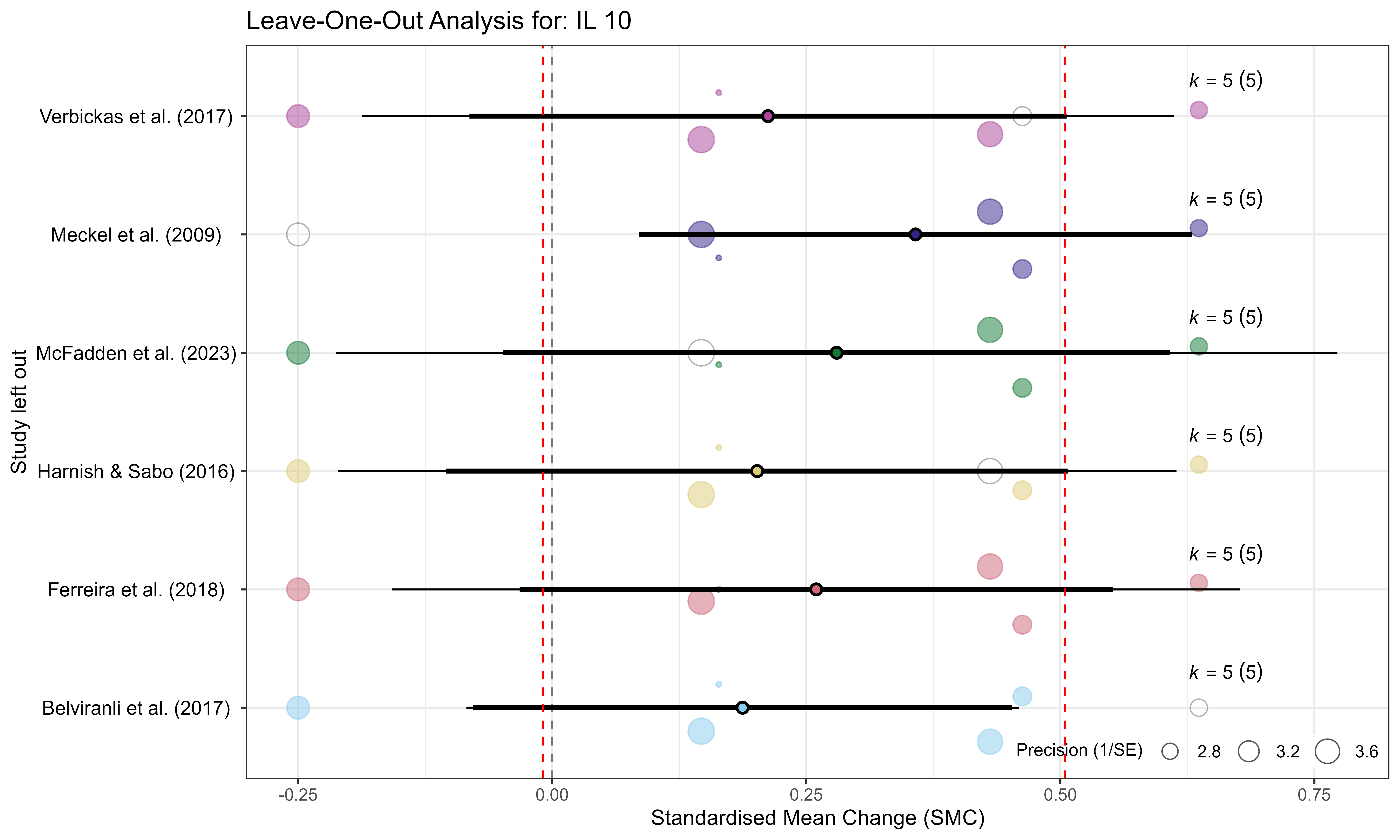

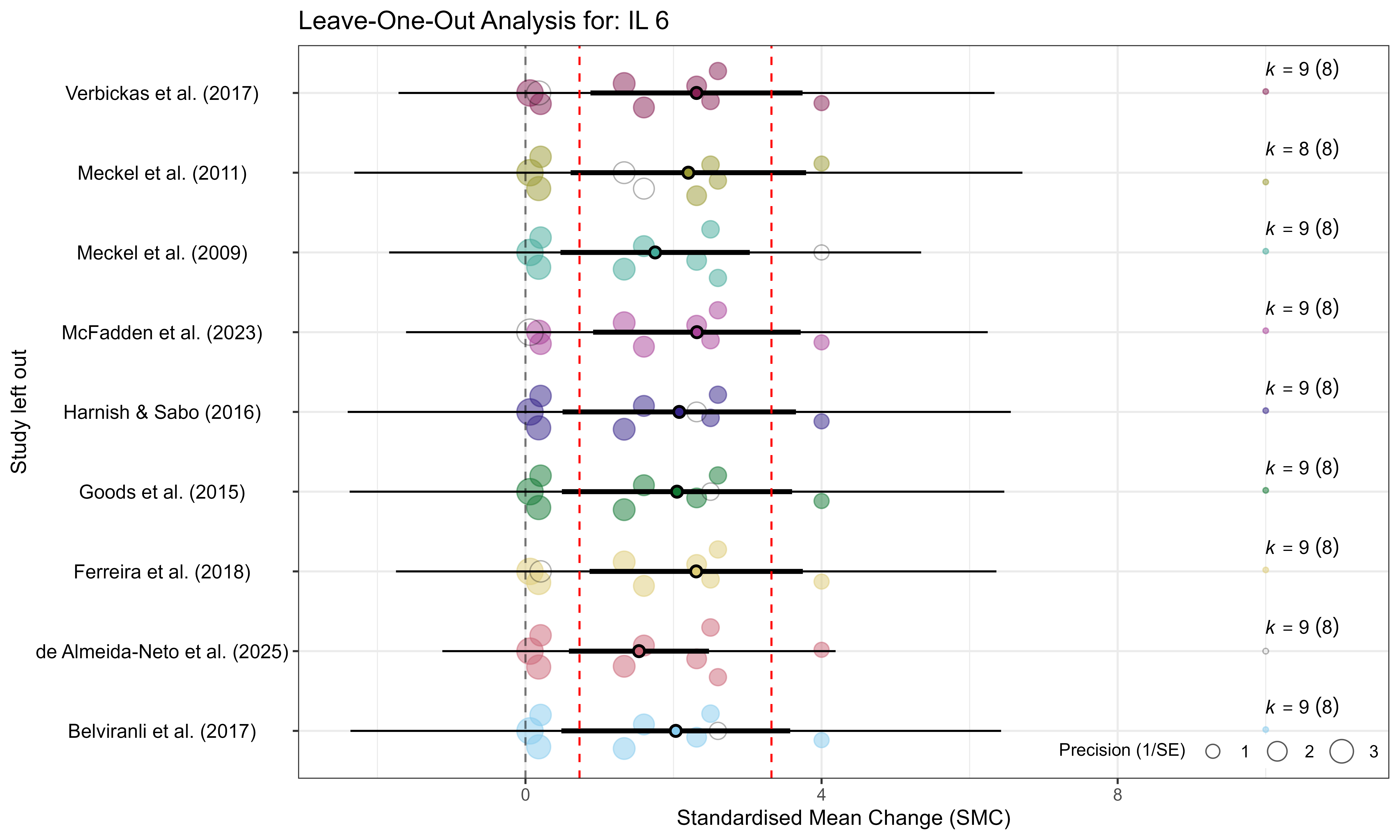

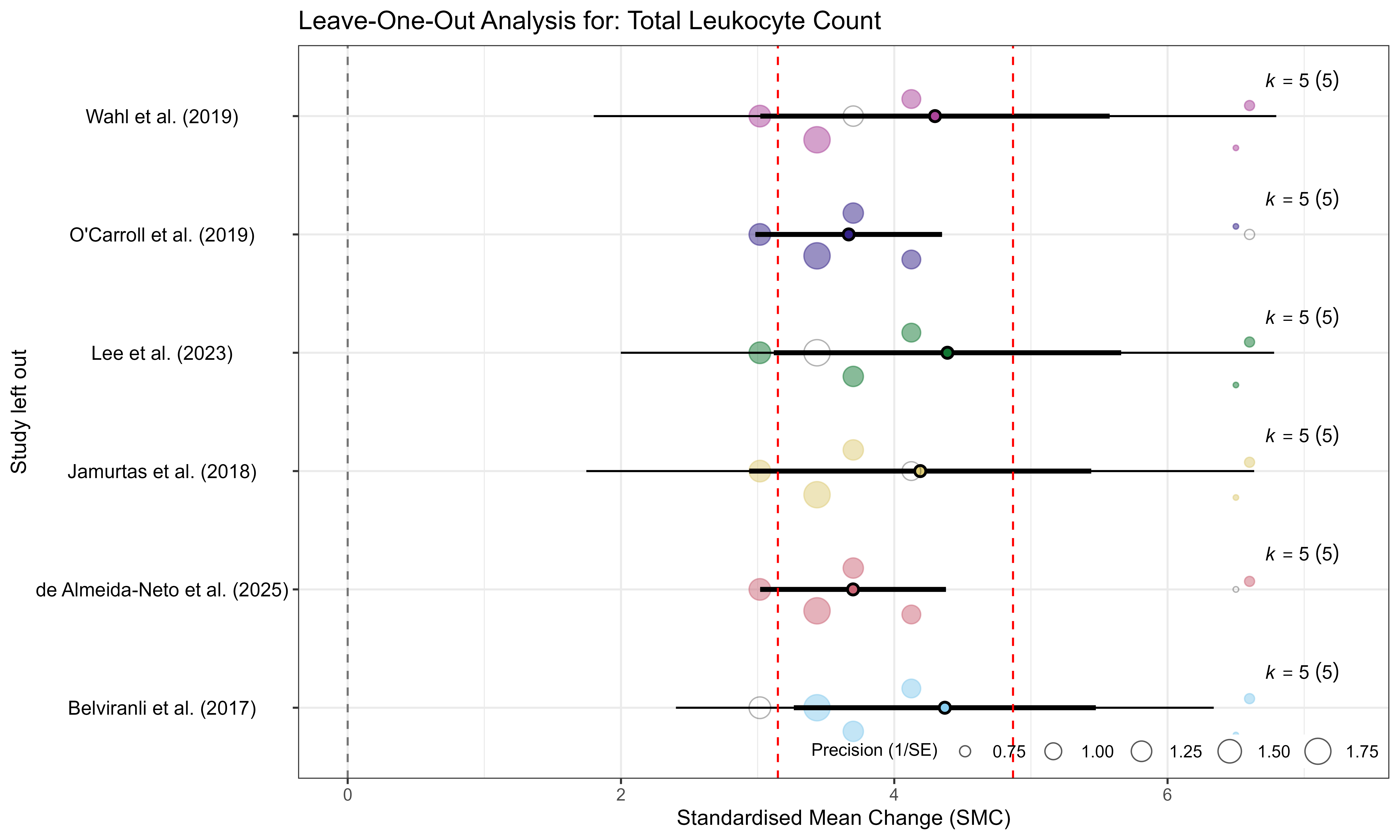

Supplement: Supplementary file 1 [file Data_Sheet_1.docx]
